# Endovascular revascularization vs. open surgical revascularization for patients with lower extremity artery disease: a systematic review and meta-analysis

**DOI:** 10.3389/fcvm.2023.1223841

**Published:** 2023-07-24

**Authors:** Hongxin Shu, Xiaowei Xiong, Xiaomei Chen, Xiaolei Sun, Rong Zhang, Ruihua Wang, Qun Huang, Jun Zhu

**Affiliations:** ^1^ The Second Clinical Medical School, Nanchang University, Nanchang, China; ^2^ Department of Vascular Surgery, The First Hospital of Nanchang, Nanchang, China; ^3^ Department of Nursing, Renji Hospital, Shanghai Jiao Tong University School of Medicine, Shanghai, China; ^4^ Department of General Surgery (Vascular Surgery), The Affiliated Hospital of Southwest Medical University, Luzhou, China; ^5^ Department of Vascular Surgery, Fengcheng Hospital, Shanghai, China; ^6^ Department of Vascular Surgery, The Affiliated Chuzhou Hospital of Anhui Medical University, Anhui, China; ^7^ Department of Vascular Surgery, The First People’s Hospital of Chuzhou, Anhui, China; ^8^ Department of Vascular Surgery, Shanghai Ninth People’s Hospital, Shanghai Jiao Tong University School of Medicine, Shanghai, China; ^9^ Department of Vascular Surgery, Lu’an Hospital of Anhui Medical University, Anhui, China; ^10^ Department of Vascular Surgery, Lu'an People's Hospital of Anhui Province, Anhui, China

**Keywords:** lower extremity artery disease, open surgery, endovascular, revascularization, meta-analysis, systematic review, peripheral artery disease

## Abstract

**Background:**

Currently, the main treatment for lower extremity artery disease (LEAD) is revascularization, including endovascular revascularization (EVR) and open surgical revascularization (OSR), but the specific revascularization strategy for LEAD is controversial. This review provided the comprehensive and recent evidence for the treatment of LEAD.

**Methods:**

Medline, Embase, and the Cochrane Library databases were searched for relevant articles. Randomized controlled trials (RCTs) and cohort studies comparing the short-term or long-term outcomes between EVR and OSR of LEAD were identified. Short-term outcomes were 30-day mortality, major amputation, wound complication, major adverse cardiovascular events (MACEs), and length of hospital stay (LOS), while long-term outcomes included overall survival (OS), amputation-free survival (AFS), freedom from re-intervention (FFR), primary patency (PP), and secondary patency (SP).

**Results:**

11 RCTs and 105 cohorts involving 750,134 patients were included in this analysis. For the pooled results of cohort studies, EVR markedly decreased the risk of 30-day mortality, wound complication, MACEs, LOS, but increased the risk of OS, FFR, PP, and SP. For the pooled outcomes of RCTs, EVR was associated with obviously lower 30-day mortality, less wound complication and shorter LOS, but higher risk of PP, and SP. However, both RCTs and cohorts did not show obvious difference in 30-day major amputation and AFS.

**Conclusions:**

Both the pooled results of cohorts and RCTs indicated that EVR was associated with a lower short-term risk for LEAD, while OSR was accompanied by a substantially lower long-term risk. Therefore, the life expectancy of LEAD should be strictly considered when choosing the revascularization modality. As the current findings mainly based on data of retrospective cohort studies, additional high-quality studies are essential to substantiate these results.

**Systematic Review Registration:**

https://www.crd.york.ac.uk/PROSPERO/#recordDetails, identifier CRD42022317239.

## Introduction

Peripheral artery disease (PAD) is currently an important global problem, affecting nearly 200 million people (1). Lower extremity artery disease (LEAD) is the manifestation of PAD in the lower extremities and is also the main disease of PAD. The primary symptom of early-stage LEAD is intermittent claudication, while rest pain and gangrene of limbs occur with the progress of LEAD (2). In 1997, the Rutherford classification was recommended to describe lower extremity ischemia and is still widely applied (3). According to this classification, the end-stage of LEAD is also termed chronic limb-threatening ischemia (CLTI), which includes ischemic rest pain and tissue loss (Rutherford 4–6). The main treatment of LEAD is revascularization. If there is no timely revascularization, 20% of CLTI patients will have to receive major amputation surgery within a year, and the overall mortality will reach to 22% (4). Therefore, timely and effective treatments are crucial for LEAD patients.

To date, endovascular revascularization (EVR) and open surgical revascularization (OSR) are the two most common options for revascularization of LEAD patients. EVR is mainly composed of percutaneous transluminal angioplasty (PTA), atherectomy, and stenting, while OSR is treated by surgical bypass and endarterectomy (5). Presently, the optimal option of revascularization is debatable. Several previous studies revealed that OSR is accompanied by low long-term mortality (6–8). Abu Dabrh et al. concluded that there is no statistical difference between EVR and OSR in long-term mortality (9). A recent meta-analysis by Wang et al. revealed that OSR reduces the long-term mortality of CLTI patients but increases the risk of short-term mortality and major adverse cardiovascular and cerebrovascular events (MACEs) (10). However, several articles fulfill the inclusion criteria have been ignored (7, 11–14), and a large number of cohort studies and randomized controlled trials (RCTs) have been published after 2018 (5, 15–17). This systematic review and meta-analysis were conducted to collect the latest evidence for the treatment of LEAD patients.

## Methods

This systematic review and meta-analysis followed the Preferred Reporting Items for Systematic Reviews and Meta-Analyses (PRISMA) and the Cochrane Handbook for Systematic Reviews of Interventions (version 5.1.0) (18). We reported observational clinical studies following the Meta-analysis of Observational Studies in Epidemiology (MOOSE) standards (19). The registration number at the International prospective register of systematic reviews (PROSPERO) was CRD42022317239 (https://www.crd.york.ac.uk/PROSPERO/#recordDetails).

### Search strategy and selection criteria

For this study, we searched Medline, Embase, and the Cochrane Library databases for original studies from inception to June, 2023. This search strategy is presented in Supplementary Material Table S1.

Inclusion criteria: (1) population: patients with lower extremity artery disease; (2) intervention and Comparison: patients were divided into EVR (such as PTA, stenting, or atherectomy) or OSR (such as bypass surgery and endarterectomy) groups; (3) outcomes: studies should report short-term (less than 30 day or in hospital) or long-term (more than 1 year); (4) study design: RCTs or cohort studies. Exclusion criteria: (1) patients with the iliac aortic disease; (2) studies written in non-English text; (3) case report, case series, letter to editor, review and animal research. No restriction was applied to the geographic regions and years of publication. We also retrieved the reference lists of relevant articles manually to broaden this search. Two independent reviewers (SH, XX) conducted this selection. Any inconsistency in study selection was settled by discussing with a third reviewer (HQ).

### Data extraction and outcomes of interest

Data were extracted by two independent reviewers (SH, XX) using a standardized Microsoft Excel file, and discrepancies were resolved by discussing with a third reviewer (HQ). The following items were extracted: the first authors' s name, year of publication, country, study design, disease stage, population characteristics, endovascular intervention, open surgery, number of patients, average age, and time of follow-up. Data were preferentially selected if shown as data after propensity-matching. The data of follow-up were shown as the maximum time-point of the Kaplan–Meier curve. For study in the absence of full text, we sent an email to authors to obtain relevant information.

The outcomes of interest included short- and long-term outcomes. 30-day and in-hospital outcomes were deemed as short-term outcomes that comprised 30-day mortality, major amputation, wound complication, MACEs, and length of hospital stay (LOS); while long-term means more than 1-year which comprised overall survival (OS), amputation-free survival (AFS), freedom from re-intervention (FFR), primary patency (PP), and secondary patency (SP) were considered as long-term outcomes.

We assessed the quality and risk of bias of enrolled studies using the Newcastle–Ottawa scale (NOS) (20) or Cochrane risk of bias tool (21). Two reviewers (HQ, SH) independently processed the assessment. For cohort studies, NOS was applied to assess quality. A cohort study with an NOS score <6 was regarded as high-risk and should be excluded from the current study. Studies with NOS scores of 6–7 and 8–9 were deemed to be at moderate risk and low risk, respectively. Regarding Cochrane Collaboration's tool, the risk of bias 2.0 tool (RoB 2.0) will be used to assess the quality of RCTs.

### Statistical analysis

An odds ratio (OR) with 95% confidence interval (CI) was utilized for binary variables. Meanwhile, weighted mean difference (WMD) with its 95% CI was applied for continuous variables. For time-to-event data, hazard ratio (HR) with standard error (SE) was obtained from original articles and pooled according to the generic inverse-variance method. If an original article did not contain HRs and SE, HRs and SE were obtained using the Tierney method (22). Due to the possible heterogeneity among the enrolled studies, the outcomes of this study were combined with the random-effects model using the Der Simonian-Laired method, and the inter-study heterogeneity was expressed with *I*^2^ values (23). For the 30-day mortality and OS, subgroup and meta-regression analyses were carried out to explore the sources of heterogeneity with respect to the following perspectives: study design (prospectively cohort, retrospective cohort), regional characteristics (Europe, America, Asia, Africa, Australia), sample size (<1,000, >1,000), and disease stage (according to information provided by the author in original studies, patients were divided into three groups: Rutherford 1–3, Rutherford 4–6, and Rutherford 1–6). For all outcomes, a leave-one-out analysis was conducted as a sensitivity analysis to explore the stability of each outcome. The funnel plots and *P*-value of Egger's test were constructed to estimate publication bias; *P*-value <0.05 of Egger's test indicated the potential publication bias (24). Trial sequential analysis (TSA) v0.9.5.10 Beta software was utilized to conduct the TSA for 30-day mortality (25, 26). The required information size (RIS) and the trial sequential monitoring boundary (TSMB) were calculated under the following conditions: relative risk reduction of 20%, the first type of error (*α* = 0.05), and power of 80%. The sample size of the accumulated evidence was sufficient when the RIS threshold was crossed by the cumulative *Z*-curve; otherwise, the sample size is inadequate and additional studies are still needed. Furthermore, the results showed significant differences if the cumulative *Z*-curve passed the TSA threshold. In this study, a *P*-value <0.05 was considered statistically significant, except for a *P-*value <0.10 in the chi-square test. All data analyses were carried out using Review Manager V5.3 (The Nordic Cochrane Centre, København, Denmark), Stata 17.0 (Stata Corp., College Station, TX, USA), and TSA v0.9.5.10 Beta software.

## Results

**Figure 1:**
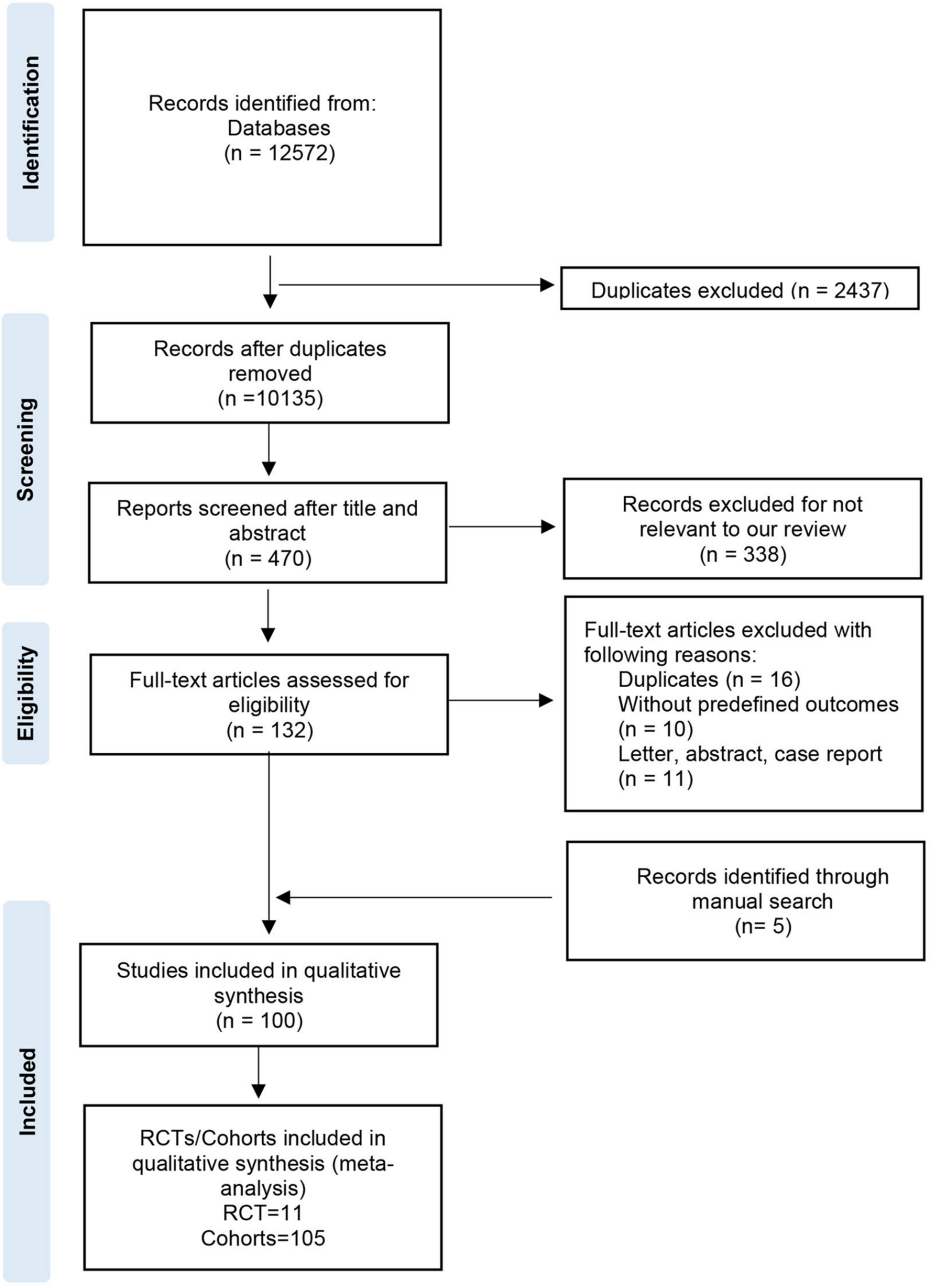
The process of studies selection.

The process of study selection was shown in Figure 1. Overall, 12,572 records were identified from databases and 5 record was retrieved by manual search; 2,437 records were excluded as these were duplicate studies. After screening the titles, abstracts, and full-text, 100 studies were included in this qualitative synthesis. Finally, a total of 116 RCTs/cohorts (11 RCTs and 105 cohorts) were enrolled in the qualitative synthesis (meta-analysis). Included articles were published between 2004 and 2023, involving 750,134 patients from Europe, America, Asia, Africa, and Australia (Supplementary Material Figure S1). Supplementary Material Table S2 showed the basic characteristics of the included studies. In the EVR group, patients received PTA, stenting, or atherectomy. In the OSR group, patients underwent open bypass surgery (autogenous, synthetic, mixed) or endarterectomy. The NOS score was 6–9 (Supplementary Material Table S3). Eleven RCTs were included in this meta-analysis. Basing on the RoB 2.0 tool, the BASIL trial, BASIL2 trial, and BEST-CLI were deemed as “low risk”; the Enzmann trial, ZILVERPASS study, Kedora study, Reijnen MMPJ 2017, Lepäntalo M 2007, McQuadeK 2009, and van der Zaag study expressed some concerns; Björkman P 2018 was regarded as “high risk” (Supplementary Material Figure S2). Among the cohort studies, the proportion of endovascular revascularization was 57%, while the proportion was 50% in RCTs.

### Short-term outcomes

**Figure 2:**
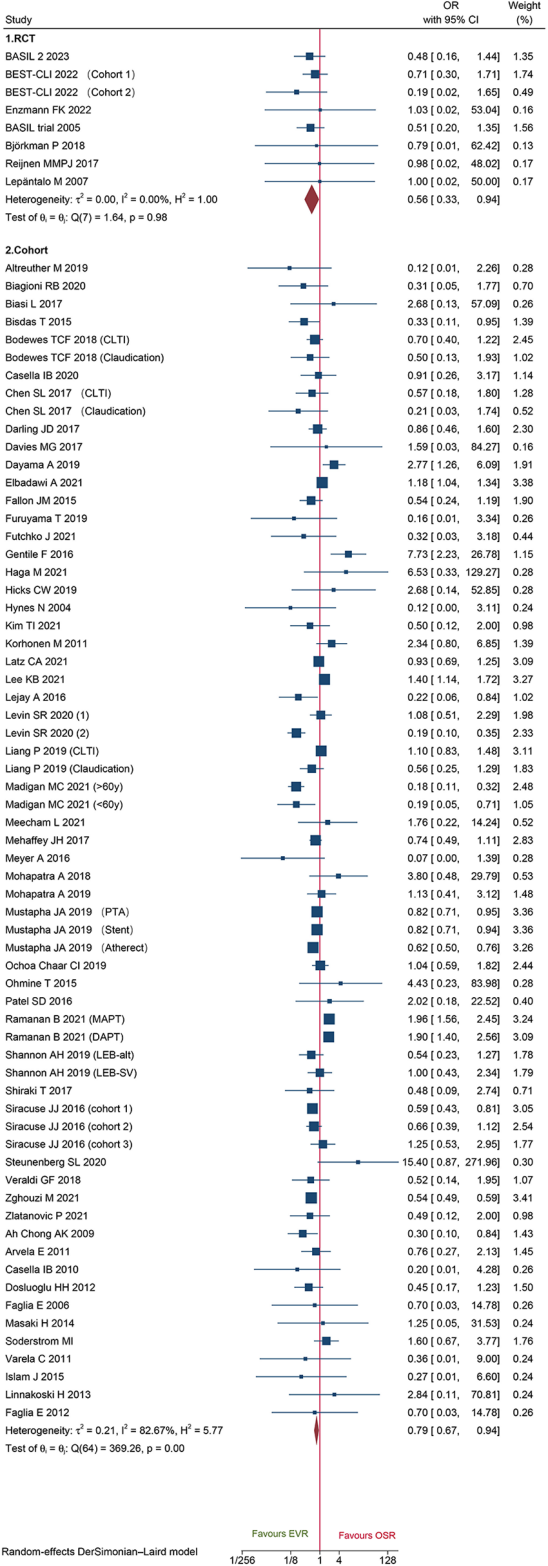
Forest plot showing the odds ratio (OR) for 30-day mortality in patients underwent endovascular revascularization (EVR) versus open surgical revascularization (OSR). CI, confidence interval; RCT, randomized controlled trial; CLTI, chronic limb-threatening ischemia; PTA, percutaneous transluminal angioplasty; MAPT, mono antiplatelet agent; DAPT, dual antiplatelet agent. Squares indicate the odds ratio, and horizontal lines represent 95% confidence intervals.

**Figure 3:**
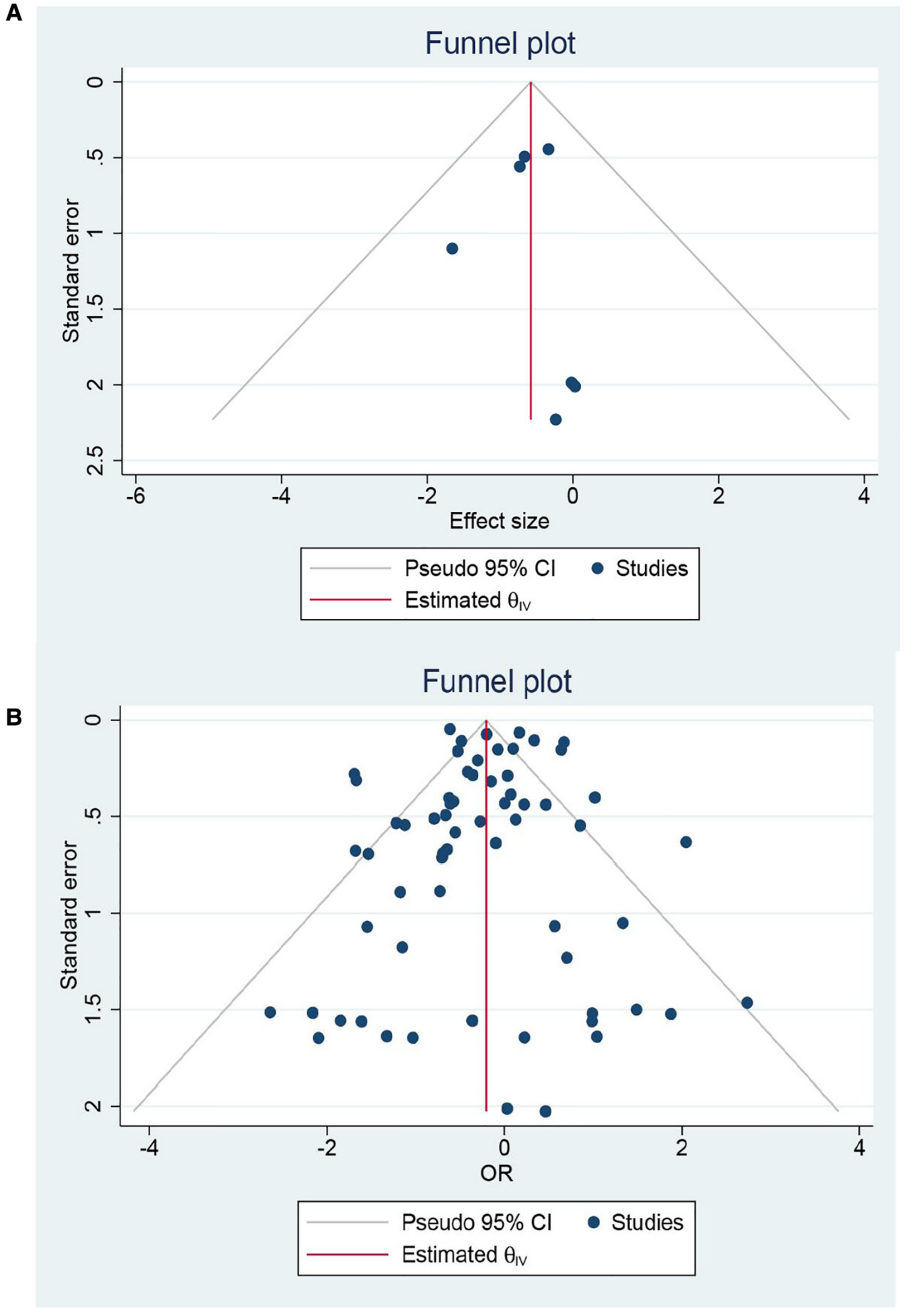
Forest plot for 30-day mortality for: (**A**) randomized controlled trials; (**B**) Cohort studies.

Eight RCTs and 65 cohorts involving 644,990 patients reported 30-day mortality. The pooled results of the cohort studies revealed that patients who received EVR had a decreased risk of 30-day death events than patients who received OSR (OR: 0.79, 95% CI: 0.67–0.94) (Figure 2). Combined data from four RCTs also found EVR had lower 30-day mortality than OSR groups (OR: 0.56, 95% CI: 0.33–0.94), which was consistent with result of cohorts. To further verify the pooled results in cohorts, we carried out TSA. The result of TSA on 30-day mortality demonstrated that the *Z*-cure crossed the TSMB, which assumed that EVR decreased the risk of 30-day mortality (Supplementary Material Figure S3). The RIS 280,865 was achieved in the current study. Subgroup analysis is presented in Supplementary Material Figure S4, indicating that the source of heterogeneity could be caused by these factors. Moreover, sensitivity analysis was conducted by discarding each study sequentially to assess the stability of outcomes. The combined outcomes were ranged from OR: 0.75 (95% CI: 0.64–0.88) to OR: 0.80 (95% CI: 0.68–0.94). The results of sensitivity analyses were consistent and revealed that the results of our study were robust. The funnel plots (Figure 3) and Egger's test (*P *= 0.9256 for RCTs; *P *= 0.4598 for cohorts) did not show any publication bias.

Herein, we identified 33 cohorts and 2 RCTs that reported 30-day major amputation and did not detect any distinct difference between EVR and OSR (OR: 0.94, 95% CI: 0.83–1.08; OR: 1.02, 95% CI: 0.52–1.97, respectively) (Supplementary Material Figure S5). In addition, sensitivity analysis was conducted by omitting each study sequentially from the cohort's pooled data. The pooled data ranged from OR: 0.93 (95% CI: 0.82–1.06) to OR: 1.00 (95% CI: 0.88–1.15), suggesting that the pooled data were consistent and stable. For cohorts, Egger's test did not detect any potential publication bias (*P *= 0.0963).

**Figure 4:**
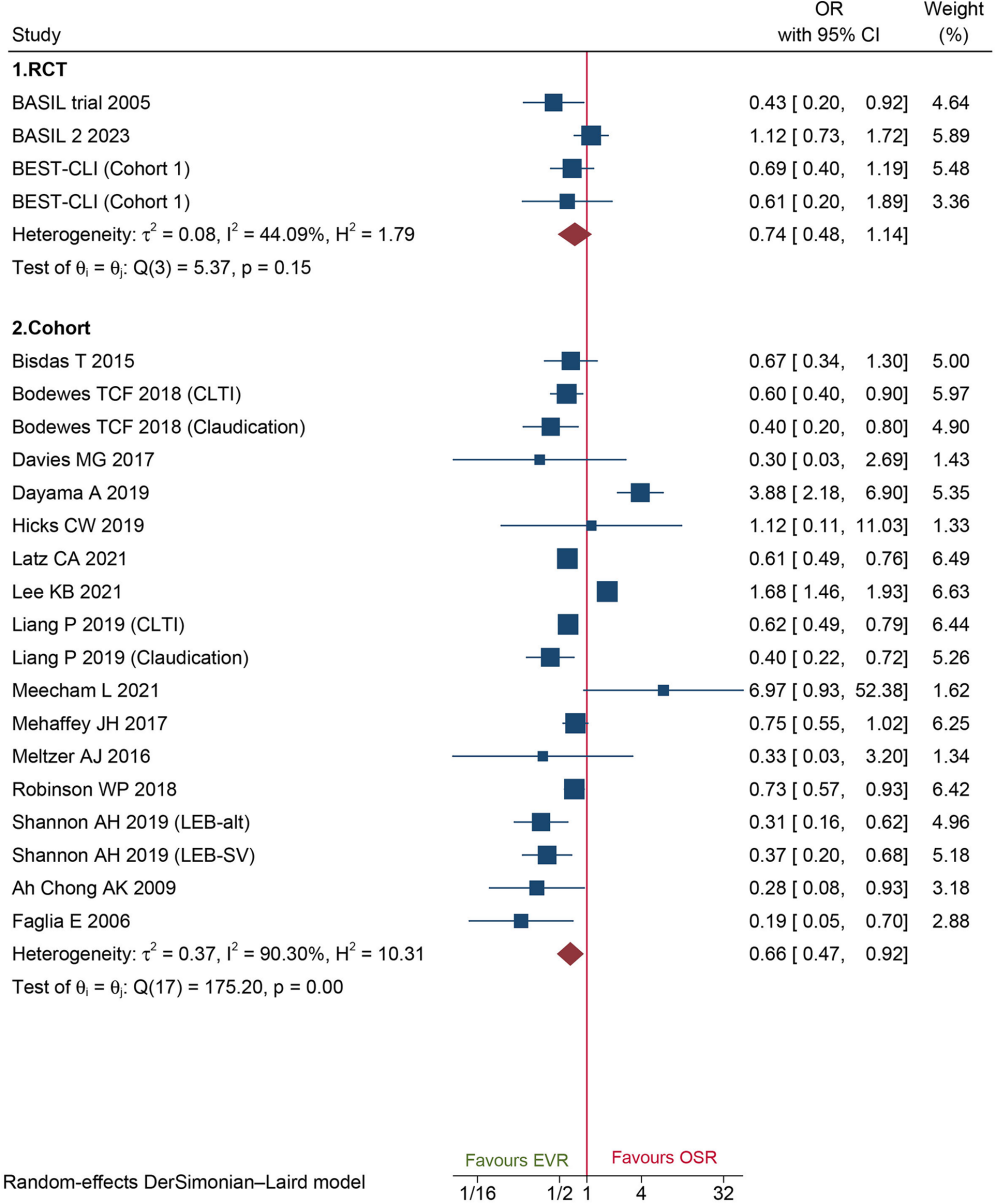
Forest plot showing the odds ratio (OR) for major adverse cardiovascular events in patients underwent endovascular revascularization (EVR) versus open surgical revascularization (OSR). CI, confidence interval; RCT, randomized controlled trial; CLTI, chronic limb-threatening ischemia. Squares indicate the odds ratio, and horizontal lines represent 95% confidence intervals.

A total of 18 cohorts and 3 RCT reported 30-day MACEs data. Data of cohorts suggested that patients who underwent EVR had a significantly lower risk of MACEs than those who underwent OSR (OR: 0.66, 95% CI: 0.47–0.92) (Figure 4). However, no significant difference was found on data of RCTs (OR: 0.74, 95%CI: 0.48–1.14) (Figure 4). Sensitivity analysis showed that the cohort's pooled data ranged from OR: 0.58 (95% CI: 0.42–0.81) to OR: 0.69 (95% CI: 0.49–0.98), indicating that the combined data were consistent and stable. No potential publication bias was found by Egger'test (*P *= 0.5034 for cohorts; *P *= 0.1419 for RCTs).

The results from 30 cohorts and 2 RCTs indicated that EVR is associated with a markedly low risk of wound complications (OR: 0.19, 95% CI: 0.10–0.37; OR: 0.34, 95% CI: 0.21–0.54, respectively) (Supplementary Material Figure S6). Sensitivity analysis showed the pooled ORs of cohort studies ranged from 0.16 (95% CI: 0.08–0.29) to 0.19 (95% CI: 0.10–0.37), suggesting that our combined outcome was consistent and stable. Potential publication bias might also not exist (Egger's test, *P *= 0.56).

**Figure 5:**
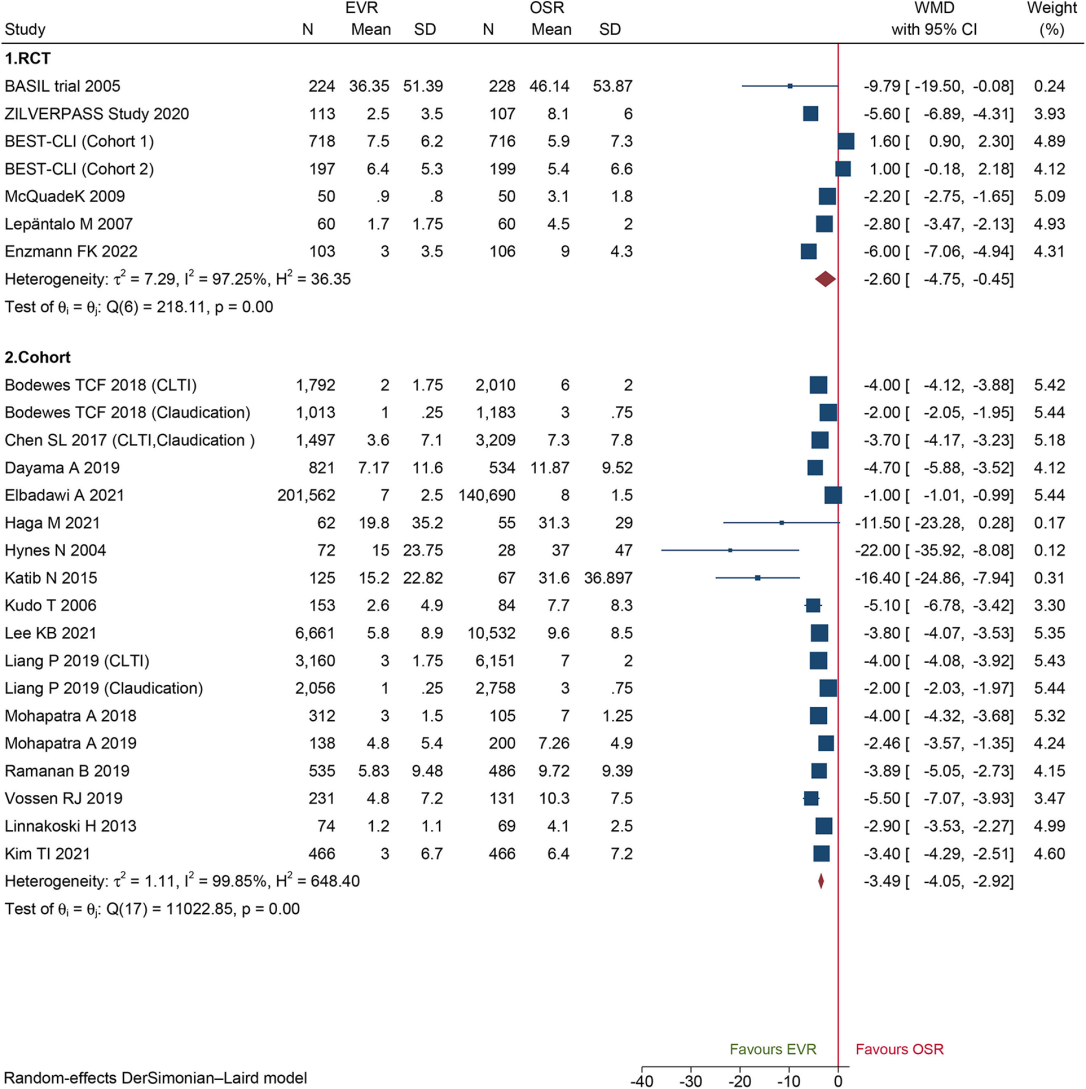
Forest plot showing the weighted mean difference (WMD) for length of hospital stay in patients underwent endovascular revascularization (EVR) versus open surgical revascularization (OSR). CI, confidence interval; RCT, randomized controlled trial; CLTI, chronic limb-threatening ischemia. Squares indicate the weighted mean difference, and horizontal lines represent 95% confidence intervals.

A total of 18 cohorts and 6 RCTs reported LOS in the hospital. Both the cohorts and RCTs data demonstrated that patients who underwent EVR had shorter LOS than those who underwent OSR (WMD −1.21, 95% CI: −1.60 to −0.82; WMD −2.60, 95% CI: −4.75 to −0.45; respectively) (Figure 5). Sensitivity analysis showed that the cohort's pooled WMD ranged from −1.26 (95% CI: −1.66 to −0.85) to −1.08 (95% CI: −1.41 to −0.75), revealing that our combined outcome was consistent and stable. We did not detect potential publication bias by Egger's test (*P *= 0.1560 for RCTs; *P *= 0.6484 for cohorts).

### Long-term outcomes

**Figure 6:**
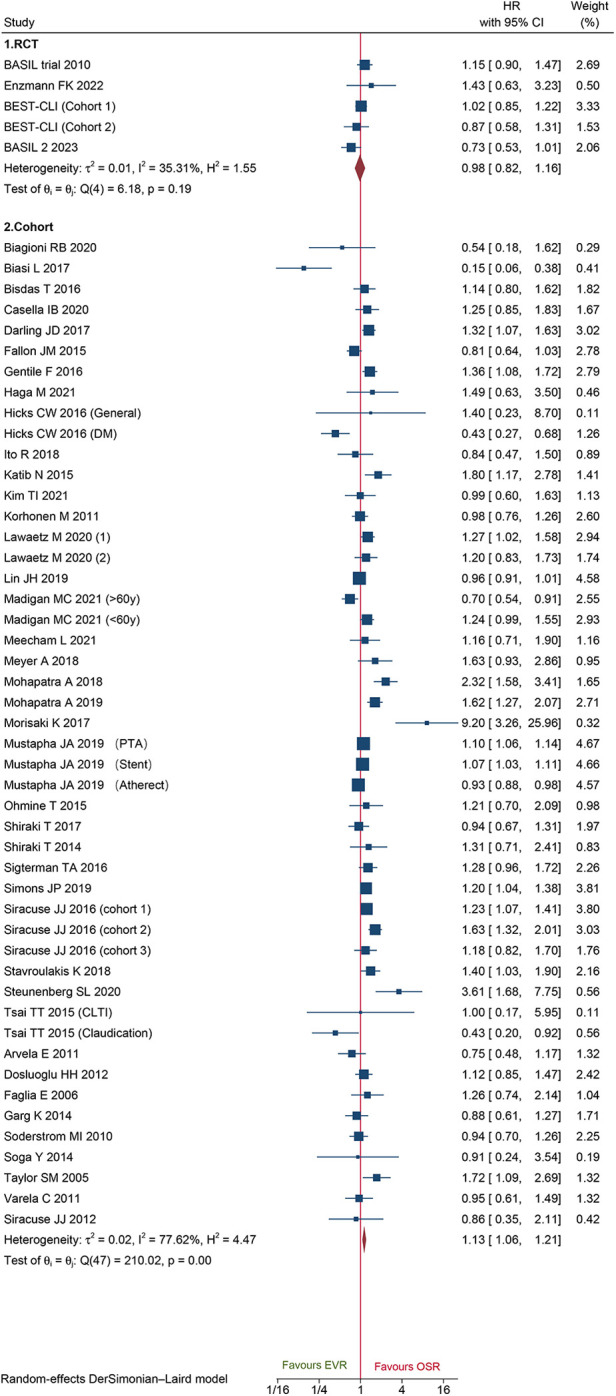
Forest plot showing the hazard ratio (HR) for over survival in patients underwent endovascular revascularization (EVR) versus open surgical revascularization (OSR). CI, confidence interval; RCT, randomized controlled trial; CLTI, chronic limb-threatening ischemia; PTA, percutaneous transluminal angioplasty; DM, diabetic mellitus. Squares indicate the hazard ratio, and horizontal lines represent 95% confidence intervals.

**Figure 7:**
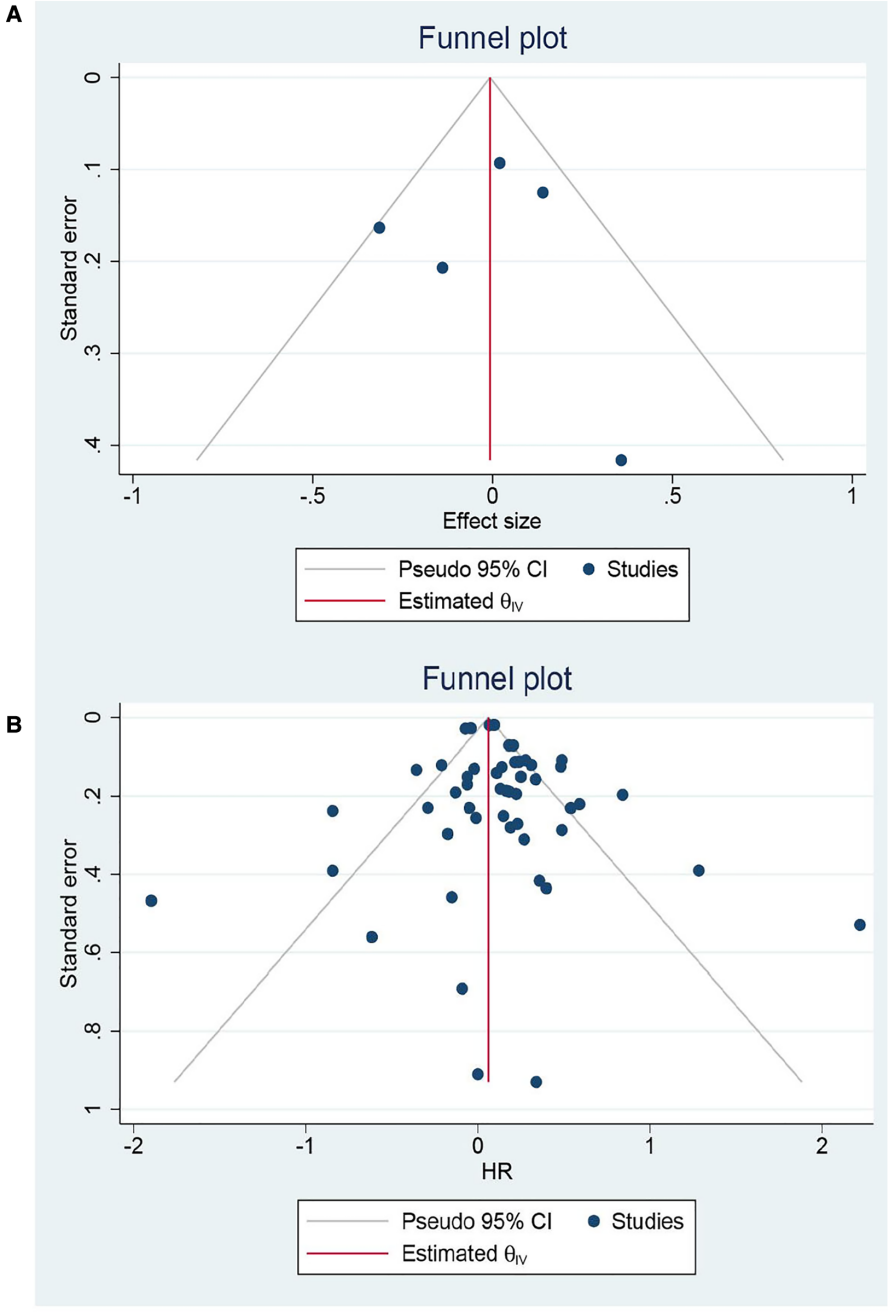
Forest plot for over survival for: (**A**) randomized controlled trials; (**B**) Cohort studies.

A total of 48 cohorts and 4 RCTs involving 132,210 patients reported OS. The combined data from cohorts demonstrated that patients who underwent EVR had a significantly higher risk of long-term death than those who received OSR (HR: 1.13, 95% CI: 1.06–1.21) (Figure 6). However, we did not observe a significant difference in RCTs (HR: 0.98, 95% CI: 0.82–1.16). Subgroup analysis revealed the source of heterogeneity across studies that could arise from the seven factors (Supplementary Material Figure S7). Sensitivity analysis of the cohort studies showed that the pooled HR ranged from 1.11 (95% CI: 1.04–1.18) to 1.14 (95% CI: 1.07–1.21), suggesting that our combined outcomes were consistent and stable. We also did not find potential publication bias under Egger's test (*P *= 0.8291 for RCTs; *P *= 0.8552 for cohorts) and funnel plots (Figure 7).

**Figure 8:**
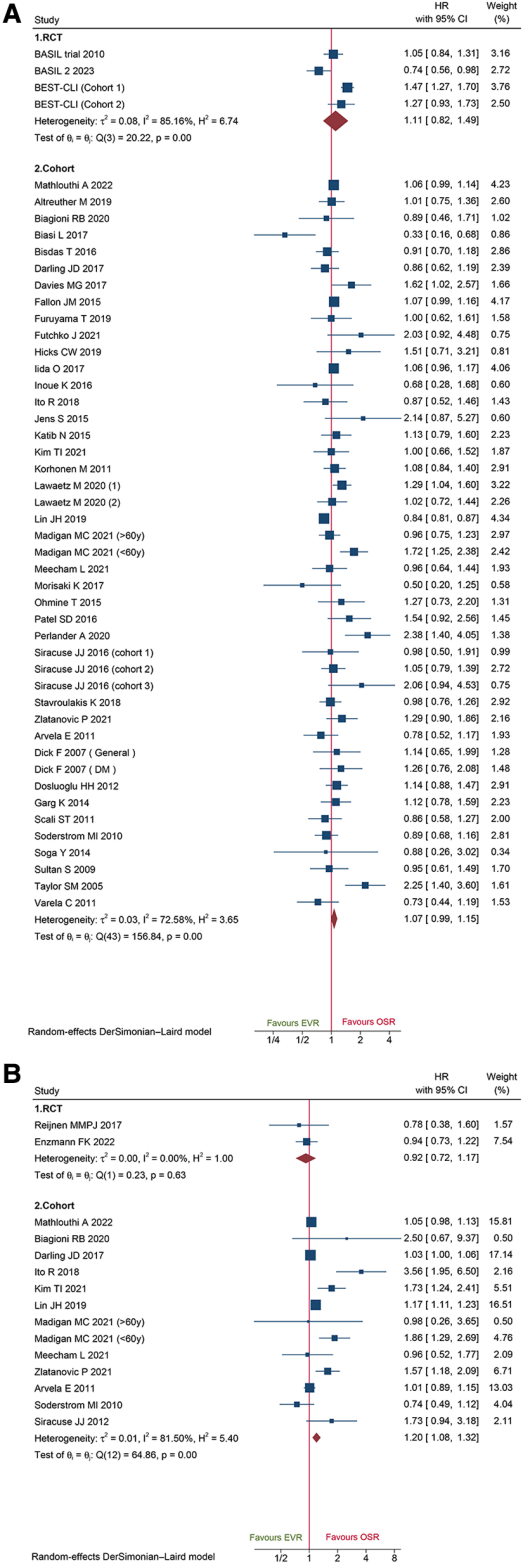
Forest plot showing the hazard ratio (HR) in patients underwent endovascular revascularization (EVR) versus open surgical revascularization (OSR) for: (**A**) amputation-free survival; (**B**) freedom from re-intervention. CI, confidence interval; RCT, randomized controlled trial; DM, diabetic mellitus. Squares indicate the hazard ratio, and horizontal lines represent 95% confidence intervals.

A total of 44 cohorts and 3 RCT recorded data on amputation-free survival. Data of cohorts and RCTs did not show any obvious difference between EVR and OSR (HR: 1.07, 95% CI: 0.99–1.15; HR: 1.11, 95%CI: 0.82–1.49) (Figure 8A). The sensitivity analysis of cohort studies showed the pooled HR from 1.06 (95% CI: 0.97–1.14) to 1.08 (95% CI: 1.01–1.17), suggesting that our pooled outcome was consistent and stable. And no potential publication bias was found by Egger'test (*P *= 0.3233 for cohorts; *P *= 0.6050 for RCTs).

Herein, we identified 2 RCTs and 13 cohorts collected from re-intervention data. Combined data of cohorts revealed that patients who underwent EVR had a markedly high risk of reintervention (HR: 1.20, 95% CI: 1.08–1.32), while pooled data of RCTs did not show any significant difference (HR: 0.92, 95%CI: 0.72–1.17) (Figure 8B). The sensitivity analysis indicated that the combined HRs ranged from 1.19 (95% CI: 1.06–1.33) to 1.36 (95% CI: 1.13–1.63), suggesting that our combined outcome was consistent and stable. And no potential publication bias was found by Egger's test (*P *= 0.3011 for cohorts).

**Figure 9:**
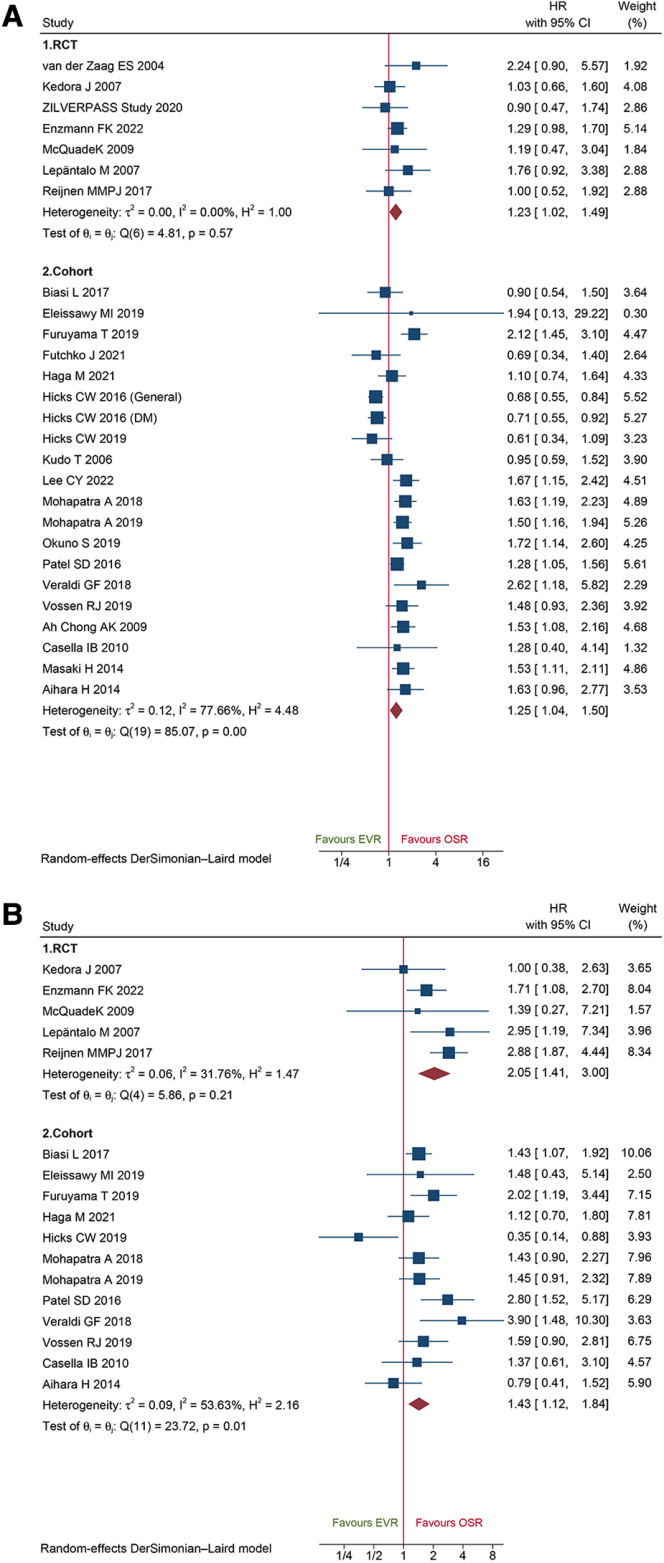
Forest plot showing the hazard ratio (HR) in patients underwent endovascular revascularization (EVR) versus open surgical revascularization (OSR) for: (**A**) primary patency; (**B**) secondary patency. CI, confidence interval; RCT, randomized controlled trial; DM, diabetic mellitus. Squares indicate the hazard ratio, and horizontal lines represent 95% confidence intervals.

A total of 20 cohort studies and 7 RCTs reported primary patency data. The pooled data from the cohorts and RCTs suggested that EVR was associated with a markedly high risk of primary patency failure (HR: 1.25, 95% CI: 1.04–1.50; HR: 1.23, 95% CI: 1.02–1.49; respectively) (Figure 9A). A total of 12 cohorts and 5 RCTs collected secondary patency data, and the combined data demonstrated that EVR increased the risk of secondary patency failure (HR: 1.43, 95% CI: 1.12–1.84; HR: 2.05, 95% CI: 1.41–3.00, respectively) (Figure 9B). Sensitivity analysis revealed that the cohort's pooled HRs for primary patency and secondary patency ranged from 1.22 (95% CI: 1.02–1.45) to 1.31 (95% CI: 1.12–1.53) and 1.33 (95% CI: 1.06–1.48) to 1.50 (95% CI: 1.24–1.82), suggesting that our combined outcome was consistent and stable. No potential publication bias was detected in primary (*P *= 0.8086 for RCTs; *P *= 0.4915 for cohorts) and secondary patency (*P *= 0.4402 for RCTs; *P *= 0.6712 for cohorts).

Meta-regression analysis was carried out to determine the significance of the study design, regional characteristics, sample size, disease stage for 30-day mortality, and OS (Supplementary Material Table S4). Only “regional characteristics” were significant (30-day mortality: *P *= 0.006; OS: *P *= 0.018).

## Discussion

Based on the pooled outcomes of cohort studies, this meta-analysis revealed that EVR has superior short-term outcomes, including 30-day mortality, 30-day wound complication, MACEs, and LOS, while OSR is associated with substantially better long-term results on OS, FFR, PP, and SP. For 30-day mortality, the cumulative z score traversed the futility area, which implied further cohort studies were not required and were unlikely to change the current conclusion. Meanwhile, the pooled outcomes of RCTs also revealed EVR is associated with better short-term outcomes, including 30-day mortality, wound complication, LOS, while OSR is associated with better long-term outcomes, including PP, SP. However, both RCTs and cohorts did not show significant difference in 30-day major amputation and AFS.

Three relevant meta-analyses were conducted on LEAD patients (9, 10, 27). The first meta-analysis by Abu Dabrh et al. revealed that EVR and OSR have similar long-term mortality (9). Some methodological concerns explained the difference in their conclusions from those of the current study. Firstly, many eligible studies were ignored, which could affect the pooled results. Secondly, study by Bergan et al. and Wolf et al. enrolled the same patients from 1 RCT (Veterans Affairs Cooperative Study, 199); therefore, their results should not be merged (28, 29). Thirdly, data from cohorts and RCTs should not be combined due to different study designs. The second meta-analysis conducted by Wang et al. revealed a conclusion similar to the current study (10). Their meta-analysis was very well written and their conclusions were valuable references to treatment of CLTI. However, their review focused only on the end-stage of LEAD, whereas the current study focused on the patients in all stages of symptomatic LEAD, as the patients with intermittent claudication should also receive revascularization when medical therapy is inadequate (30). Notably, some eligible studies were not included in their review, which would affect the merged outcomes, our study is the most comprehensive. Meanwhile, patients in the study Dosluoglu 2009 (31) and Dosluoglu 2012 (32) were reduplicative and should not be simultaneously included. Furthermore, the exclusion criteria of Wang et al. did not contain iliac aortic diseases, while the present only focused on the occlusion of lower limb arteries, which is emphasized in the Methods section. The TSA method was performed in the current study compared to these two previous reviews. The required sample size was estimated to be 279,101, and the cumulative *Z* curve met the required sample size and conventional threshold that affirmed the validity of our study. Recently, an individual participant data (IPD) meta-analysis, conducted by Farhan S et al, also revealed that EVR was associated with less early complications and shorter length of hospital stay than OSR, which was accordant with the current analysis (27).

The methods of revascularization for LEAD have always been controversial. Recent guidelines indicated that the trend of EVR for LEAD had increased markedly in recent years with the advances in endovascular technology and its characteristics of minimally invasive (33). Thus, some surgeons advocated EVR as the first choice for LEAD, while OSR is the second option (33). However, EVR is usually accompanied by injury to vascular endothelial cells and smooth muscle cells (VSMCs), which promote the proliferation of VSMCs and cause restenosis (34, 35). For OSR therapy, adequate inflow and outflow and an appropriate autogenous vein are essential but are not easily obtained in many end-stage LEAD patients (30). Therefore, comprehensive evidence is an urgent requirement for the treatment of symptomatic LEAD patients. The current review concluded that life expectancy is a critical factor and should be considered when choosing the revascularization modality.

Since heterogeneity was observed in the present review, sensitivity analyses, meta-regression, and subgroup analyses were performed. Sensitivity analyses revealed that our combined data were stable and consistent. The results of meta-regression indicated that regional characteristics might be the potential source of heterogeneity. The subgroup analyses conducted according to the regional characteristics revealed that our 30-day mortality and OS data were mainly from the America. Reasonably, the pooled results of America subgroup were approximated to the whole group. A previous study also found that racial differences lead to variations in LEAD risk and presentation (36). Therefore, these results should be interpreted carefully as most studies were conducted in the America, and future studies should focus on the influence of regional characteristics on the prognosis of LEAD.

Nevertheless, the present study has some limitations. Firstly, only 11 RCTs were enrolled, and most of the included cohort studies were retrospective, which might cause a selection bias. The current study also showed that some combined results of RCTs and cohorts were different; as only 11 RCTs were included, more high-quality RCTs are essential. Secondly, substantial heterogeneity was noted across studies, but the sensitivity analyses revealed that our pooled data were stable and consistent. Finally, we did not define the specific modalities of EVR and OSR as inconvenient to obtain the original data.

## Conclusion

Overall, the results of cohort studies revealed that EVR is associated with lower short-term risk, including 30-day mortality, wound complication, MACEs, and LOS but higher long-term risk, such as OS, FFR, PP, and SP. Meanwhile, the results of RCTs were consistent with cohort studies in 30-day mortality, wound complication, LOS, PP and SP, which further validated the reliability of above results. Life expectancy is a critical factor for determining the specific revascularization method. For elderly patients, EVR seems to be an appropriate option, while OSR seems to be a more suitable treatment for younger patients. However, as the current findings are mainly based on retrospective cohort studies, high-quality studies are required to validate our conclusion.

## Data Availability

The original contributions presented in the study are included in the article/**Supplementary Material**, further inquiries can be directed to the corresponding authors.
