# Supplementary material for: Endovascular revascularization vs. open surgical revascularization for patients with lower extremity artery disease: a systematic review and meta-analysis
Source: Front Cardiovasc Med. 2023 Jul 24;10:1223841. doi: 10.3389/fcvm.2023.1223841 (PMC10405177; doi:10.3389/fcvm.2023.1223841)
Supplement: Supplementary file 1 [file Datasheet1.docx]

**Supplemental material**

**E****ndovascular revascularization versus open surgical revascularization for patients with lower extremity artery disease: a systematic review and meta-analysis**

| Index | | |
| --- | --- | --- |
| Supplemental Table S1 | Literature search strategy | Page 02 |
| Supplemental Table S2 | The characteristics of the included studies | Page 03 |
| Supplemental Table S3 | Quality assessment of included cohort studies | Page 18 |
| Supplemental Table S4 | The results of meta- regression | Page 22 |
| Supplemental Figure S1 | The distribution of included studies in published year, geographical location | Page 23 |
| Supplemental Figure S2 | Risk of bias assessment of the included RCTs | Page 24 |
| Supplemental Figure S3 | The trial sequential analysis results of 30-day mortality | Page 25 |
| Supplemental Figure S4 | Subgroup analysis of 30-day mortality | Page 26 |
| Supplemental Figure S5 | Pooled results of 30-day major amputation | Page 27 |
| Supplemental Figure S6 | Pooled results of wound complication | Page 28 |
| Supplemental Figure S7 | Subgroup analysis of overall survival | Page 29 |
| Supplemental References |  | Page 30 |

**Supplemental Table S1. Literature search strategy.**

| #1 | Revascularization[Title/Abstract] |
| --- | --- |
| #2 | Surgical revascularization[Title/Abstract] |
| #3 | Open intervention[Title/Abstract] |
| #4 | Open Procedures[Title/Abstract] |
| #5 | Open therapy[Title/Abstract] |
| #6 | Bypass[Title/Abstract] |
| #7 | #1 OR #2 OR #3 OR #4 OR #5 OR #6 |
| #8 | Endovascular intervention[Title/Abstract] |
| #9 | Endovascular revascularization[Title/Abstract] |
| #10 | Endovascular Procedures[MeSH Terms] |
| #11 | Endovascular therapy[Title/Abstract] |
| #12 | Endovascular[Title/Abstract] |
| #13 | Angioplasty[Title/Abstract] |
| #14 | #8 OR #9 OR #10 OR #11 OR #12 OR #13 |
| #15 | Arteriosclerosis obliterans[Title/Abstract] |
| #16 | Atherosclerosis obliterans[Title/Abstract] |
| #17 | Arteriosclerosis obliterans[MeSH Terms] |
| #18 | Peripheral Arterial Disease[MeSH Terms] |
| #19 | Limb ischemia[Title/Abstract] |
| #20 | Extremity ischemia[Title/Abstract] |
| #21 | #15 OR#16 OR #17 OR #18 OR #19 OR #20 |
| #22 | #7 AND #14 AND #21 |

**Supplemental Table S2. The characteristics of the included studies**

| Study/Cohort (year) | Country | Design | Multicenter | Disease stage | Population Characteristics | Endovascular revascularization‖ | Open surgery revascularization‖ | Outcomes Assessed | Matching/ Adjustment of Baseline | Number of patients | | Average age, year¶ | | Follow-up time, month | NOS score |
| --- | --- | --- | --- | --- | --- | --- | --- | --- | --- | --- | --- | --- | --- | --- | --- |
|  |  |  |  |  |  |  |  |  |  | EVR | OSR | EVR | OSR |  |  |
| BASIL 2 2023(1) | UK, Sweden, Denmark | RCT | Yes | Rutherford 4-6 | General | PTA | Autogenous bypass surgery | 30-day mortality, MACE, AFS, OS | - | 173 | 172 | 72.5 (62·7–79·7) | 72·4 (64·3–78·7) | 40 | - |
| BEST-CLI 2022 (Cohort 1)(2) | United States, Canada, Finland, Italy, and New Zealand | RCT | Yes | Rutherford 4-6 | General | Any available endovascular technique | Greater saphenous vein | 30-day mortality, MACE, LOS, AFS, OS | - | 716 | 718 | 67.0±10.0 | 66.9±9.8 | 2.7 years | - |
| BEST-CLI 2022 (Cohort 2)(2) | United States, Canada, Finland, Italy, and New Zealand | RCT | Yes | Rutherford 4-6 | General | Any available endovascular technique | Alternative bypass conduit | 30-day mortality, MACE, LOS, AFS, OS | - | 199 | 197 | 68.8±9.6 | 68.4±8.8 | 1.6 years | - |
| Enzmann FK 2022(3) | Austria | RCT | No | Rutherford 3-6 | General | Stenting | Autogenous bypass surgery | 30-day mortality, PP, SP OS | - | 103 | 106 | 69.3 ± 7.0 | 68.5 ± 7.8 | 48 | - |
| ZILVERPASS Study 2020(4) | Belgium | RCT | Yes | Rutherford 1-6 | General | Stenting | Dacron/PTFE bypass surgery | PP, LOS | - | 113 | 107 | 69.6 ± 10.8 | 67.6 ± 10.1 | 12 | - |
| Björkman P 2018(5) | Finland | RCT | Yes | Rutherford 1-4 | General | Stenting | Prosthetic bypass surgery | 30-day mortality | - | 23 | 18 | 68 ± 10 | 67 ± 8.5 | 24 | - |
| Reijnen MMPJ 2017(6) | Netherlands | RCT | Yes | Rutherford 3-6 | General | Heparin-Bonded Endoluminal | Mixed bypass surgery | 30-day mortality, FFR, PP, SP | - | 63 | 62 | 68.5 ± 8.8 | 66.7 ± 7.9 | 12 | - |
| McQuade K 2009(7) | USA | RCT | No | Rutherford 1-6 | General | Stenting | Prosthetic bypass surgery | LOS, PP, SP | - | 50 | 50 | 72 ± 9.9 | 67 ± 10.7 | 24 | - |
| BASIL trial 2010(8) | UK | RCT | Yes | Rutherford 1-6 | General | PTA | Mostly autogenous bypass surgery | OS, AFS | - | 224 | 228 | NR | NR | 63.6 (36-92.6) † | - |
| BASIL trial 2005(9) | UK | RCT | Yes | Rutherford 1-6 | General | PTA | Mostly autogenous bypass surgery | 30-day mortality, 30-day amputation, MACEs, wound complication, LOS | - | 224 | 228 | NR | NR | 60 | - |
| Kedora J 2007(10) | USA | RCT | No | Rutherford 1-6 | General | Stenting, PTA | Prosthetic bypass surgery | PP, SP | - | 40 | 46 | 71.8 ± 9.9 | 66.9 ± 10.7 | 12 | - |
| Lepäntalo M 2009(11) | Finland | RCT | Yes | Rutherford 1-6 | General | Thrupass endograft | Polytetrafluoroethylene bypass | 30-day mortality, LOS, PP, SP | - | 60 | 60 | 64 ± 7.75 | 66 ± 6.75 | 36 | - |
| van der Zaag ES 2004(12) | Netherlands | RCT | Yes | Rutherford 1-4 | General | Stenting, PTA | Autogenous bypass surgery | PP, 30-day major amputation, wound complication | - | 31 | 25 | 68 (45–84) | 66 (42–83) | 26-28 | - |
| Mathlouthi A 2022(13) | USA | Retrospective cohort | No | Rutherford 4-6 | General | Stenting, PTA | Mixed bypass surgery | AFS, AFF | NA | 12062 | 5166 | 73.2 ± 11.3 | 72.3 ± 9.9 | 33 | 7 |
| Lee CY 2022(14) | Taiwan | Retrospective cohort | No | Rutherford 4-6 | General | Stenting, PTA | Mixed bypass surgery | PP | NA | 94 | 93 | 75.8 ± 13.4 | 74.4 ± 10.3 | 36 | 7 |
| Elbadawi A 2021(15) | USA | Retrospective cohort | Yes | Rutherford 4-6 | DM | Stenting, PTA, atherectomy | Mixed bypass surgery | 30-day mortality, 30-day major amputation, wound infection, LOS | Baseline-adjusted | 141724 | 140342 | 68.67 ± 11.67 | 68.18 ± 11.20 | 1 | 8 |
| Futchko J 2021(16) | USA | Retrospective cohort | No | Rutherford 4-6 | General | Stenting | Non-autogenous bypass surgery | AFS, PP, 30-day mortality, LOS | Baseline-adjusted | 65 | 60 | 68 | 73 | 60 | 8 |
| Haga M 2021(17) | Japan | Retrospective cohort | No | Rutherford 4-6 | General | PTA | Autogenous bypass surgery | OS, PP, SP, 30-day mortality, incisional complication, LOS | NA | 62 | 55 | 75.8 ± 11.5 | 72.1 ± 8.7 | 24 | 7 |
| Kim TI 2021(18) | USA | Retrospective cohort | Yes | Rutherford 1-6 | General | Stenting, PTA, atherectomy | Mixed bypass surgery bypass surgery | OS, AFS, FFR, 30-day mortality, 30-day major amputation, wound complication, LOS | Baseline-adjusted | 466 | 466 | NR | NR | 67.2 | 8 |
| Latz CA 2021(19) | USA | Retrospective cohort | Yes | Rutherford 4-6 | General | Stenting, PTA | Mixed bypass surgery | 30-day mortality, 30-day major amputation, MACEs | Propensity Score Analysis | 4780 | 6003 | 70.2 ± 12.0 | 68.6 ± 11.4 | 1 | 7 |
| Lee KB 2021(20) | USA | Retrospective cohort | Yes | Rutherford 4-6 | General | Stenting | Mixed bypass surgery | 30-day mortality, 30-day major amputation, MACEs, wound complication, LOS | Baseline-adjusted | 10532 | 6661 | 69.6 ± 12.0 | 68.4 ± 11.4 | 1 | 7 |
| Madigan MC 2021 (>60y)(21) | USA | Retrospective cohort | Yes | Rutherford 1-6 | General | NR | NR | OS, AFS, FFR, 30-day mortality | Propensity Score Analysis | 2041 | 1021 | 67.1 ± 5.6 | 67.2 ± 5.5 | 1 | 7 |
| Madigan MC 2021 (<60y)(21) | USA | Retrospective cohort | Yes | Rutherford 1-6 | General | NR | NR | OS, AFS, FFR, 30-day mortality | Propensity Score Analysis | 1697 | 851 | 52.9 ± 5.2 | 52.9 ± 4.7 | 1 | 7 |
| Meecham L 2021(22) | UK | Retrospective cohort | No | Rutherford 4-6 | General | PTA | Mixed bypass surgery | OS, AFS, FFR, 30-day mortality, 30-day major amputation, MACEs | NA | 234 | 45 | 76.9 ± 11.1 | 70.0 ± 9.9 | 84 | 8 |
| Ramanan B 2021 (MAPT)(23) | USA | Retrospective cohort | Yes | Rutherford 4-6 | General | NR | NR | 30-day mortality, 30-day major amputation | NA | 13374 | 10572 | 69.58±12.09 | 67.64±11.32 | 12 | 7 |
| Ramanan B 2021 (DAPT)(23) | USA | Retrospective cohort | Yes | Rutherford 4-6 | General | NR | NR | 30-day mortality, 30-day major amputation | NA | 20407 | 6537 | 68.18±11.68 | 66.84±10.84 | 12 | 7 |
| Zghouzi M 2021(24) | USA | Retrospective cohort | Yes | Rutherford 1-6 | General | Angioplasty, atherectomy, stenting | Endarterectomy, bypass | 30-day mortality, 30-day major amputation | Baseline-adjusted | 99279 | 69141 | 69.95 ± 11.998 | 76.22 ± 11.486 | 1 | 7 |
| Zlatanovic P 2021(25) | Serbia | Retrospective cohort | Yes | Rutherford 4-6 | General | PTA, stenting | Mixed bypass surgery | AFS, FFR, 30-day mortality, 30-day major amputation, wound complication, | Propensity-matching | 235 | 235 | 68.54 ± 7.07 | 67.67 ± 7.69 | 60 | 8 |
| Biagioni RB 2020(26) | Brazil | Retrospective cohort | Yes | Rutherford 4-5 | General | Stenting, PTA | Autogenous bypass surgery | OS, AFS, 30-day mortality | Demographic data | 65 | 43 | 66.9 ± 20.7 | 68.1 ± 9.4 | 36 | 8 |
| Casella IB 2020(27) | Brazil | Retrospective cohort | No | Rutherford 4-7 | General | Stenting | Autogenous bypass surgery | 30-day mortality, OS | NA | 105 | 48 | 68.9 ± 10.8 | 63.9 ± 11.1 | 36 | 7 |
| Lawaetz M 2020 (1)(28) | Denmark | Retrospective cohort | No | Rutherford 4-6 | General | Stenting, PTA | Autogenous bypass surgery | OS, AFS | Comorbidities | 239 | 363 | 74 | 70 | 84 | 8 |
| Lawaetz M 2020 (2)(28) | Denmark | Retrospective cohort | No | Rutherford 4-6 | General | Stenting, PTA | Synthetic bypass surgery | OS, AFS | Comorbidities | 239 | 77 | 74 | 73 | 84 | 8 |
| Levin SR 2020 (1)(29) | USA | Retrospective cohort | Yes | Rutherford 1-3 | General | Stenting, PTA | Mixed bypass surgery | 30-day mortality, wound complication | NA | 7051 | 2527 | 66.7 ± 10.1 | 64.8 ± 10.3 | 12 | 7 |
| Levin SR 2020 (2)(29) | USA | Retrospective cohort | Yes | Rutherford 1-3 | General | Stenting, PTA | Mixed bypass surgery | 30-day mortality, wound complication | NA | 7051 | 849 | 66.7 ± 10.1 | 60.7 ± 9.4 | 12 | 7 |
| Perlander A 2020(30) | Sweden | Prospectively cohort | Yes | Rutherford 4-6 | General | Stenting | Synthetic bypass surgery | AFS | Baseline-adjusted | 117 | 73 | 75 | 73 | 24 | 8 |
| Steunenberg SL 2020(31) | Netherlands | Prospectively cohort | Yes | Rutherford 4-6 | General | Stenting | Endarterectomy, mixed bypass | OS, 30-day mortality | NA | 82 | 67 | 81 | 76 | 12 | 7 |
| Altreuther M 2019(32) | Norway | Retrospective cohort | No | Rutherford 4-6 | General | Stenting, PTA | Autogenous bypass surgery | AFS, 30-day mortality, 30-day major amputation, 30-day complications | NA | 107 | 129 | 75 | 74 | 12 | 7 |
| Dayama A 2019(33) | Germany | Retrospective cohort | Yes | Rutherford 4-6 | General | Stenting | Mixed bypass surgery | MACEs, 30-day major amputation, 30-day mortality, wound complication, LOS | Baseline-adjusted | 821 | 534 | 68.61 ± 11.42 | 66.64 ± 12.20 | 1 | 7 |
| Eleissawy MI 2019(34) | Egypt | Prospectively cohort | No | Fontaine IIb, III, IV | General | Stenting, PTA | Mixed bypass surgery | PP, SP, wound infection | NA | 28 | 25 | 72 ± 12.1 | 72 ± 7.5 | 12 | 7 |
| Furuyama T 2019(35) | Japan | Retrospective cohort | No | Rutherford 4-6 | General | Stenting, PTA | Prosthetic grafts bypass surgery | AFS, PP, SP, 30-day mortality | NA | 85 | 69 | 74.9 ± 1.15 | 74.7 ± 1.27 | 36 | 8 |
| Hicks CW 2019(36) | USA | Retrospective cohort | Yes | Rutherford 4-6 | DM | Stenting, PTA | Mostly autogenous bypass surgery | PP, SP, AFS, superficial surgical site infections, MACEs, 30-day mortality, 30-day major amputation | Baseline-adjusted | 142 | 53 | NR | NR | 48 | 7 |
| Liang P 2019 (CLTI)(37) | USA | Retrospective cohort | Yes | Rutherford 4-6 | General | Stenting, PTA | Mixed bypass surgery | 30-day mortality, wound complication, MACEs, LOS | NA | 3160 | 6151 | NR | NR | 1 | 7 |
| Liang P 2019 (Claudication)(37) | USA | Retrospective cohort | Yes | Rutherford 1-3 | General | Stenting, PTA | Mixed bypass surgery | 30-day mortality, wound complication, MACEs, LOS | NA | 2056 | 2758 | NR | NR | 1 | 7 |
| Lin JH 2019(38) | USA | Retrospective cohort | Yes | Rutherford 4-6 | General | NR | NR | OS, AFS, FFR | Patient ability, hospital revascularization experience, and propensity score weighting | 10830 | 5970 | 70 ± 12 | 71 ± 12 | 80 | 8 |
| Mohapatra A 2019(39) | USA | Retrospective cohort | No | Rutherford 4-6 | General | Stenting, PTA, Atherectomy | Mixed bypass surgery | OS, 30-day mortality, 30-day major amputation, wound complication, PP, SP, LOS | NA | 138 | 200 | 76.1 ± 11.4 | 74.1 ± 12.3 | 48 | 9 |
| Mustapha JA 2019 (PTA)(40) | USA | Retrospective cohort | Yes | Rutherford 4-6 | General | PTA | Mixed bypass surgery | OS, 30-day mortality | Propensity Score Analysis | 10677 | 10310 | 74 ± 12 | 72 ± 10 | 48 | 8 |
| Mustapha JA 2019 (Stent)(40) | USA | Retrospective cohort | Yes | Rutherford 4-6 | General | Stenting | Mixed bypass surgery | OS, 30-day mortality | Propensity Score Analysis | 11429 | 10310 | 74 ± 11 | 72 ± 10 | 48 | 8 |
| Mustapha JA 2019 (Atherect)(40) | USA | Retrospective cohort | Yes | Rutherford 4-6 | General | Atherectomy | Mixed bypass surgery | OS, 30-day mortality | Propensity Score Analysis | 4434 | 10310 | 74 ± 11 | 72 ± 10 | 48 | 8 |
| Ochoa Chaar CI 2019(41) | USA | Retrospective cohort | Yes | Rutherford 4-6 | General | Atherectomy, Angioplasty, Stenting | Autogenous bypass surgery | 30-day mortality | NA | 1410 | 1668 | 71.3 ± 0.5 | 68.4 ± 0.5 | 12 | 7 |
| Okuno S 2019(42) | Japan | Retrospective cohort | Yes | Rutherford 2-6 | General | Stenting, subintimal angioplasty | Autogenous bypass surgery | PP | NA | 260 | 92 | 73.6 ± 8.9 | 71.0 ± 8.5 | 22.0 ± 21.4 | 8 |
| Ramanan B 2019(43) | USA | Retrospective cohort | Yes | Rutherford 4-6 | HD | Angioplasty, atherectomy, stenting | Endarterectomy, autogenous vein bypass | 30-day mortality, 30-day major amputation, wound complication, LOS | Demographics, baseline comorbidities | 535 | 486 | 65.80 ± 11.03 | 66.22 ± 10.83 | 1 | 7 |
| Shannon AH 2019 (LEB-alt)(44) | USA | Prospectively cohort | Yes | Rutherford 4-6 | General | NR | Mixed bypass surgery | 30-day mortality, 30-day major amputation, MACEs, surgical site infection | NA | 683 | 314 | 70 ±12.3 | 68.4 ±11.4 | 1 | 7 |
| Shannon AH 2019 (LEB-SV)(44) | USA | Prospectively cohort | Yes | Rutherford 4-6 | General | NR | Autogenous bypass surgery | 30-day mortality, 30-day major amputation, MACEs, surgical site infection | NA | 683 | 570 | 70 ±12.3 | 67.6 ±11.6 | 1 | 7 |
| Simons JP 2019(45) | USA | Retrospective cohort | Yes | Rutherford 4-6 | General | Angioplasty, atherectomy, stenting, PTA | Autogenous bypass surgery | OS | Baseline-adjusted | 24124 | 14256 | NR | NR | 60 | 8 |
| Vossen RJ 2019(46) | Netherlands | Retrospective cohort | No | Fontaine II, III, IV | General | PTA, stenting | Autogenous bypass surgery | PP, SP, LOS | Propensity-matched analysis | 103 | 103 | NR | NR | 120 | 8 |
| Bodewes TCF 2018 (CLTI)(47) | Netherlands | Retrospective cohort | Yes | Rutherford 4-6 | General | NR | NR | 30-day mortality, 30-day amputation, MACEs, Surgical site infection, LOS | Baseline-adjusted | 1792 | 2010 | NR | NR | 1 | 7 |
| Bodewes TCF 2018 (Claudication)(47) | Netherlands | Retrospective cohort | Yes | Rutherford 1-3 | General | NR | NR | 30-day mortality, 30-day amputation, MACEs, Surgical site infection, LOS | Baseline-adjusted | 1013 | 1183 | NR | NR | 1 | 7 |
| Ito R 2018(48) | Japan | Retrospective cohort | Yes | Rutherford 4-6 | HD | Mostly PTA | Autogenous bypass surgery | AFS, FFR, OS | Baseline-adjusted | 126 | 128 | 71 ± 10 | 68 ± 9 | 32 | 8 |
| Meyer A 2018(49) | Germany | Retrospective cohort | No | Rutherford 4-6 | ESRD | PTA | Mixed bypass surgery | OS | NA | 42 | 35 | NR | NR | 24 | 7 |
| Mohapatra A 2018(50) | USA | Retrospective cohort | No | Rutherford 4-6 | General | Stenting, PTA, Atherectomy | Mixed bypass surgery | OS, AFS, 30-day mortality, 30-day major amputation, wound complication, PP, SP, LOS | NA | 312 | 105 | 74.0 ± 11.5 | 70.5 ± 12.1 | 60 | 9 |
| Robinson WP 2018(51) | USA | Retrospective cohort | Yes | Rutherford 4-6 | General | Atherectomy, stenting | Mixed bypass surgery | 30-day major amputation, MACEs | NA | 3526 | 3833 | NR | NR | 1 | 7 |
| Stavroulakis K 2018(52) | Germany | Prospectively cohort | Yes | Rutherford 4-6 | General | NR | NR | AFS, OS | NA | 642 | 284 | 75 | 73 | 30 | 7 |
| Veraldi GF 2018(53) | Italy | Retrospective cohort | No | Rutherford 1-5 | General | Stenting, PTA | Autogenous bypass surgery | PP, SP, 30-day mortality, 30-day major amputation | NA | 40‡ | 40‡ | 76 | 72 | 26.7 (3-46) † | 8 |
| Biasi L 2017(54) | UK | Prospectively cohort | No | Rutherford 4-6 | Octogenarian, nonagenarian | Stenting, PTA | Autogenous bypass surgery | OS, AFS, PP, SP, 30-day mortality | NA | 79 | 41 | 85 ± 5 | 85 ± 4 | 24 | 8 |
| Chen SL 2017 (CLTI)(55) | USA | Retrospective cohort | Yes | Rutherford 4-6 | Active smokers | Stenting | NR | 30-day mortality, 30-day major amputation, wound infection, LOS | Demographics, perioperative variables, postoperative outcomes defined | 833 | 2212 | NR | NR | 1 | 7 |
| Chen SL 2017 (Claudication)(55) | USA | Retrospective cohort | Yes | Rutherford 1-3 | Active smokers | Stenting | NR | 30-day mortality, 30-day major amputation, wound infection, LOS | Demographics, perioperative variables, postoperative outcomes defined | 664 | 997 | NR | NR | 1 | 7 |
| Darling JD 2017(56) | USA | Retrospective cohort | No | Rutherford 4-6 | General | Stenting | Mixed bypass surgery | OS, AFS, FFR, 30-day mortality | NA | 668 | 668 | 70.8 ± 12.5 | 72.3 ± 12.7 | 36 | 8 |
| Davies MG 2017(57) | USA | Retrospective cohort | No | Rutherford 1-4 | General | Stenting | Autogenous bypass surgery | AFS, 30-day mortality, MACEs, 30-day major amputation | NA | 40 | 64 | 75 ± 15 | 68 ± 13 | 36 | 8 |
| Iida O 2017(58) | Japan | Prospectively cohort | Yes | Rutherford 4-6 | General | Stenting | Autogenous bypass surgery | AFS | Baseline-adjusted | 295 | 149 | 73 ± 9 | 72 ± 9 | 36 | 8 |
| Mehaffey JH 2017(59) | USA | Retrospective cohort | Yes | Rutherford 4-6 | General | Stenting, atherectomy | Mixed bypass surgery | 30-day mortality, 30-day major amputation, MACEs | Propensity Score Analysis | 1924 | 1924 | 69 ± 12 | 69 ± 12 | 1 | 7 |
| Morisaki K 2017(60) | Japan | Retrospective cohort | No | Rutherford 4-6 | General | Stenting, PTA | Autogenous bypass surgery | OS, AFS | NA | 31 | 85 | 71.5 ± 1.5 | 71.8 ± 1.1 | 24 | 8 |
| Shiraki T 2017(61) | Japan | Retrospective cohort | Yes | Rutherford 4-6 | HD | Stenting, PTA | Autogenous bypass surgery | OS, 30-day mortality | Propensity-matching | 63 | 63 | 69 ±10 | 69 ±9 | 21 (8-33) † | 8 |
| Bisdas T 2016(62) | Germany | Prospectively cohort | Yes | Rutherford 4-6 | General | Mixed | Mixed bypass surgery | AFS, OS | Baseline-adjusted | 642 | 284 | 75 | 73 | 24 | 8 |
| Gentile F 2016(63) | Sweden | Prospectively cohort | No | Rutherford 4-6 | General | Stenting, PTA | Autogenous bypass surgery | OS, 30-day mortality, wound complication | Age, lesion characteristics, comorbidities | 430 | 110 | 77 | 74 | 60 | 8 |
| Hicks CW 2016 (General)(64) | USA | Retrospective cohort | Yes | Rutherford 4-6 | General | Stenting, PTA | Mostly autogenous bypass surgery | OS, PP | Baseline-adjusted | 2066 | 500 | 76 (65-84)* | 67 (54-77)* | 12 | 7 |
| Hicks CW 2016 (DM)(64) | USA | Retrospective cohort | Yes | Rutherford 4-6 | DM | Stenting, PTA | Mostly autogenous bypass surgery | OS, PP | Baseline-adjusted | 1818 | 355 | 68 (60-76)* | 68 (60-76)* | 12 | 7 |
| Inoue K 2016(65) | Japan | Retrospective cohort | No | Rutherford 5 | General | NR | NR | AFS | NA | 25 | 21 | 71 ± 2 | 77 ± 2 | 36 | 7 |
| Lejay A 2016(66) | France | Retrospective cohort | No | Rutherford 4-6 | Nonagenarians | Stenting, PTA | Autogenous bypass surgery | 30-day mortality | NA | 116 | 73 | 93.4 | 72.1 | 10.38 | 7 |
| Meltzer AJ 2016(67) | USA | Retrospective cohort | Yes | Rutherford 4-6 | General | Stenting | Mixed bypass surgery | MACEs | Propensity Score Analysis | 113 | 113 | 69.4 ± 11.8 | 70.5 ± 12.3 | 12 | 7 |
| Meyer A 2016(68) | Germany | Prospectively cohort | Yes | Rutherford 4-6 | General | Stenting | Mixed bypass surgery | 30-day mortality, 30-day major amputation | NA | 391 | 198 | NR | NR | 60 | 7 |
| Patel SD 2016(69) | UK | Retrospective cohort | No | Rutherford 4-6 | General | Stenting, PTA | Autogenous bypass surgery | AFS, PP, SP, 30-day mortality | Propensity Score Analysis | 125 | 125 | 73 ± 11 | 74 ± 10 | 20 ± 14 | 8 |
| Sigterman TA 2016(70) | Netherlands | Retrospective cohort | No | Rutherford 4-6 | General | NR | Mixed bypass surgery | OS | Age, lesion characteristics, comorbidities | 209 | 81 | 74 (43-96)* | 72 (40-90)* | 12 | 7 |
| Siracuse JJ 2016 (cohort 1)(71) | USA | Retrospective cohort | Yes | Rutherford 4-6 | General | NR | Mostly autogenous bypass surgery | OS, AFS, 30-day mortality | Baseline-adjusted | 4838 | 3059 | 70.7 ± 12.1 | 68.1 ± 11.5 | 36 | 7 |
| Siracuse JJ 2016 (cohort 2)(71) | USA | Retrospective cohort | Yes | Rutherford 4-6 | Lacked comorbidities | NR | Mostly autogenous bypass surgery | OS, AFS, 30-day mortality | Baseline-adjusted | 2452 | 2127 | 72.3 ± 11.9 | 67.7 ± 11.8 | 36 | 7 |
| Siracuse JJ 2016 (cohort 3)(71) | USA | Retrospective cohort | Yes | Rutherford 4-6 | Required only a superficial femoral artery intervention or femoral-to-above-knee popliteal artery bypass. | NR | Mostly autogenous bypass surgery | OS, AFS, 30-day mortality | Baseline-adjusted | 507 | 502 | 70.5 ± 11.8 | 66.4 ±11.3 | 36 | 7 |
| Bisdas T 2015(72) | Germany | Prospectively cohort | Yes | Rutherford 4-6 | General | Mixed | Mixed bypass surgery | 30-day mortality, 30-day amputation, MACEs | NA | 642 | 284 | 75 | 73 | 24 | 7 |
| Fallon JM 2015(73) | USA | Retrospective cohort | Yes | Rutherford 1-6 | HD | PTA | Mixed bypass surgery | OS, AFS, 30-day mortality | NA | 394 | 295 | NR | NR | 24 | 8 |
| Jens S 2015(74) | Netherlands | Prospectively cohort | No | Fontaine III, IV | General | Stenting, PTA | Mostly autogenous bypass surgery | AFS, wound complication | NA | 86 | 27 | 71.2 ± 11.4 | 63.5 ± 11.3 | 12 | 6 |
| Katib N 2015(75) | Australia | Retrospective cohort | No | Rutherford 4-6 | General | Stenting, PTA | Mostly autogenous bypass surgery | OS, AFS, LOS | Age, comorbidities | 125 | 67 | 75 | 73.2 | 35.1 ± 31.9 | 8 |
| Ohmine T 2015(76) | Japan | Retrospective cohort | No | Rutherford 4-6 | General | NR | Autogenous bypass surgery | OS, AFS, 30-day mortality, 30-day major amputation | Age, comorbidities | 102 | 48 | 76 | 72.3 | 24 | 8 |
| Tsai TT 2015 (CLTI)(77) | USA | Retrospective cohort | Yes | Rutherford 4-6 | General | NR | NR | OS | Propensity-matching | 174 | 174 | 73.1 ± 10.7 | 73.0 ± 9.6 | 33.6** | 8 |
| Tsai TT 2015 (Claudication)(77) | USA | Retrospective cohort | Yes | Rutherford 1-3 | General | NR | NR | OS | Propensity-matching | 162 | 162 | 66.0 ± 9.6 | 66.3 ± 10.3 | 43.2** | 8 |
| Islam J 2015(78) | South Africa | Retrospective cohort | No | Rutherford 4-6 | General | Stenting, PTA | Mostly polytetrafluoroethylene grafts bypass surgery | 30-day mortality | NA | 118 | 95 | 67 | 65 | 10 | 8 |
| Shiraki T 2014(79) | Japan | Retrospective cohort | Yes | Rutherford 4-6 | General | Stenting, PTA | Autogenous bypass surgery | OS | Age, lesion characteristics, comorbidities | 396 | 63 | 72 ± 10 | 72 ± 8 | 24 | 8 |
| Garg K 2014(80) | USA | Retrospective cohort | No | Rutherford 4-6 | General | Stenting, PTA | Mixed bypass surgery | OS, AFS | Age, comorbidities | 187 | 105 | 75.5 ± 11.6 | 74.1 ± 11.5 | 60 | 7 |
| Masaki H 2014(81) | Japan | Retrospective cohort | No | Fontaine III, IV | General | PTA | Mostly autogenous bypass surgery | PP, 30-d mortality, wound complications, MACEs | Age, lesion characteristics, comorbidities | 31 | 119 | 72 (47–89) † | 70 (46–89)† | 60 | 8 |
| Soga Y 2014(82) | Japan | Retrospective cohort | Yes | Rutherford 4-6 | General | Stenting, PTA | Autogenous bypass surgery | OS, AFS | Propensity-matching | 200 | 200 | 72.0 ± 11.0 | 71.9 ± 9.5 | 30 ± 16 | 8 |
| Aihara H 2014(83) | Japan | Retrospective cohort | Yes | Rutherford 1-3 | General | Stenting, PTA | Mixed bypass surgery | PP, SP | NA | 177 | 86 | 73±8 | 71±8 | 30 ± 25 | 7 |
| Linnakoski H 2013(84) | Finland | Retrospective cohort | No | Rutherford 1-6 | General | Stenting, PTA | Mostly polytetrafluoroethylene grafts | 30-day mortality, 30-day major amputation, wound complication, LOS | NA | 68 | 63 | 72 ±10.0 | 74 ±8.8 | 16-18 | 6 |
| Dosluoglu HH 2012(85) | USA | Retrospective cohort | No | Rutherford 4-6 | General | Stenting, PTA | Mixed bypass surgery | OS, AFS, 30-d mortality, wound complication | Age, lesion characteristics, comorbidities | 295 | 138 | 73.0 ±10.5 | 69.2 ± 11.2 | 28.4 ± 23.1 | 7 |
| Siracuse JJ 2012(86) | USA | Retrospective cohort | No | NR | General | Stenting, PTA | NR | OS, FFR, wound complication | NA | 105 | 113 | 69 ±11.3 | 63 ±11.2 | 48 | 7 |
| Faglia E 2012(87) | Italy | Prospectively cohort | No | Rutherford 4-6 | DM | Stenting, PTA | Mixed bypass surgery | 30-day mortality, 30-day major amputation | NA | 292 | 40 | 72.7± 9.2 | 72.9±10.0 | 12 | 7 |
| Korhonen M 2011(88) | Finland | Retrospective cohort | No | Rutherford 4-6 | General | Stenting, PTA | Mostly autogenous bypass surgery | OS, AFS, 30-day mortality | Propensity Score Analysis | 241 | 241 | 74.3 ± 11.8 | 72.7 ± 10.2 | 31.2 ± 26.4 | 9 |
| Arvela E 2011(89) | Finland | Retrospective cohort | No | Rutherford 4-6 | Octogenarian | Stenting, PTA | Mostly autogenous bypass surgery | OS, AFS, FFR, 30-d mortality | Propensity-matching | 95 | 95 | 85.8(4.0) | 85.3(3.6) | 24 ± 25.2 | 9 |
| Scali ST 2011(90) | USA | Retrospective cohort | No | Rutherford 3-6 | General | Stenting | Autogenous bypass surgery | AFS | Comorbidities | 82 | 103 | 70 ± 11 | 66 ± 22 | 36 | 7 |
| Varela C 2011(91) | Spain | Retrospective cohort | No | Rutherford 4-6 | General | NR | NR | OS, AFS,30-d mortality | Age, lesion characteristics, comorbidities | 42 | 46 | NR | NR | 10.3 ± 9.5 | 9 |
| Casella IB 2010(92) | Brazil | Retrospective cohort | No | Rutherford 4-6 | General | Stenting, PTA | Autogenous bypass surgery | PP, SP, 30-d mortality, MACEs, wound complications | Age, lesion characteristics, comorbidities | 44 | 49 | 71.45 ± 8.63 | 70.64 ± 8.97 | 16.5 (1–84) † | 8 |
| Soderstrom MI 2010(93) | Finland | Retrospective cohort | No | Rutherford 4-6 | General | Stenting, PTA | Mostly autogenous bypass surgery | OS, AFS, FFR, 30-d mortality | Propensity-matching | 208 | 208 | 74.3 ± 11.3 | 73.7 ± 11.0 | 31.2±26.4 | 9 |
| Ah Chong AK 2009(94) | China | Retrospective cohort | No | Rutherford 4-6 | General | NR | NR | PP, 30-d mortality, MACEs, wound complications, LOS | Lesion characteristics, comorbidities | 100 | 314 | 77 | 74 | 36 | 7 |
| Sultan S 2009(95) | Ireland | Retrospective cohort | No | Rutherford 4-6 | General | Subintimal angioplasty | Mostly autogenous bypass surgery | AFS | Age, lesion characteristics, comorbidities | 190 | 119 | 73±13 | 70±14 | 60 | 8 |
| Dick F 2007 (General)(96) | Sweden | Prospectively cohort | No | Rutherford 4-6 | General | Stenting, PTA | Mixed bypass surgery | AFS | Lesion characteristics | 88 | 60 | NR | NR | 12 | 7 |
| Dick F 2007 (DM)(96) | Sweden | Prospectively cohort | No | Rutherford 4-6 | DM | Stenting, PTA | Mixed bypass surgery | AFS | Lesion characteristics | 119 | 25 | NR | NR | 12 | 7 |
| Kudo T 2006(97) | USA | Retrospective cohort | No | Rutherford 4-6 | General | Stenting, PTA | Mixed bypass surgery | PP, LOS | Age, lesion characteristics, comorbidities | 153 | 84 | 70.2 ± 12.4 | 68.3 ± 10.8 | 23 (1–122) † | 8 |
| Faglia E 2006(98) | Italy | Retrospective cohort | No | Rutherford 4-6 | DM | Stenting, PTA | Mostly autogenous bypass surgery | OS, 30-d mortality, major amputation, MACEs, wound complication | Age, lesion characteristics, comorbidities | 420 | 117 | 72.7 ± 9.2 | 72.9 ±10.0 | 40.8±15.6 | 8 |
| Taylor SM 2005(99) | USA | Retrospective cohort | No | Rutherford 4-6 | Octogenarian | Stenting, PTA | Autogenous bypass surgery | OS, AFS | NA | 65 | 57 | NR | NR | 36 | 6 |
| Hynes N 2004(100) | Ireland | Prospectively cohort | No | Rutherford 4-6 | General | Subintimal angioplasty | NR | 30-day mortality, LOS | NA | 74 | 28 | 71 | 73 | 15.0 ± 0.6 | 7 |

RCT, randomized controlled trial; NR, not reported; NA, not available; OS, overall survival; AFS, amputation-free survival; FFR, freedom from reintervention; PP, primary patency; SP, secondary patency; MACEs, major adverse cardiovascular events; LOS, Length of hospital stay; CLTI, chronic limb-threatening ischemia; EVR, endovascular revascularization; OSR, open surgical revascularization; DM, diabetes mellitus; HD, hemodialysis-dependent; PTA, percutaneous transluminal angioplasty; LEB-alt, lower extremity bypass with alternative conduit; LEB-SV, lower extremity bypass with single saphenous vein. ESRD, end-stage renal disease; MAPT, mono antiplatelet agent; DAPT, dual antiplatelet agent; PTFE, polytetrafluoroethylene.

The cohort of Levin SR 2020 (1) included patients with infrainguinal disease, while Levin SR 2020 (2) included patients with suprainguinal disease.

*, data was presented as median IQR.

**, data were presented as median.

†, data were presented as median (range).

‡, the number of limbs.

¶, data were presented as mean ± SD.

‖, Mixed revascularization strategies mean multiple endovascular (or open) methods were conducted.

**Supplemental Table S3. Quality assessment of included cohort studies.**

| Study | Representativeness of the exposed cohort | Selection of nonexposed cohort | Ascertainment of exposure | Absence of outcome at the start of the study | Comparability of cohorts | Assessment of outcome | Length of follow-up | Adequacy of follow-up | NOS score |
| --- | --- | --- | --- | --- | --- | --- | --- | --- | --- |
| Mathlouthi A 2022 | * | * | * | * | - | * | * | * | 7 |
| Lee CY 2022 | * | * | * | * | - | * | * | * | 7 |
| Elbadawi A 2021 | * | * | * | * | ** | * | - | * | 8 |
| Futchko J 2021 | * | * | * | * | * | * | * | * | 8 |
| Haga M 2021 | * | * | * | * | * | - | * | * | 7 |
| Kim TI 2021 | * | * | * | * | * | * | * | * | 8 |
| Latz CA 2021 | * | * | * | * | * | * | - | * | 7 |
| Lee KB 2021 | * | * | * | * | * | * | * | - | 7 |
| Madigan MC 2021 | * | * | * | * | * | * | * | - | 7 |
| Meecham L 2021 | * | * | * | * | * | * | * | * | 8 |
| Ramanan B 2021 | * | * | * | * | * | * | - | * | 7 |
| Zghouzi M 2021 | * | * | * | * | * | * | - | * | 7 |
| Zlatanovic P 2021 | * | * | * | * | * | * | * | * | 8 |
| Biagioni RB 2020 | * | * | * | * | * | * | * | * | 8 |
| Casella IB 2020 | - | * | * | * | * | * | * | * | 7 |
| Lawaetz M 2020 | * | * | * | * | * | * | * | * | 8 |
| Levin SR 2020 | * | * | * | * | * | * | * | - | 7 |
| Perlander A 2020 | * | * | * | * | * | * | * | * | 8 |
| Steunenberg SL 2020 | * | * | * | * | * | - | * | * | 7 |
| Altreuther M 2019 | * | * | * | * | * | * | * | - | 7 |
| Dayama A 2019 | * | * | * | * | * | * | - | * | 7 |
| Eleissawy MI 2019 | * | * | * | * | * | * | - | * | 7 |
| Furuyama T 2019 | * | * | * | * | * | * | * | * | 8 |
| Hicks CW 2019 | * | * | * | * | * | - | * | * | 7 |
| Liang P 2019 | * | * | * | * | * | * | * | - | 7 |
| Lin JH 2019 | * | * | * | * | * | * | * | * | 8 |
| Mohapatra A 2019 | * | * | * | * | ** | * | * | * | 9 |
| Mustapha JA 2019 | * | * | * | * | * | * | * | * | 8 |
| Ochoa Chaar CI 2019 | * | * | * | * | * | * | - | * | 7 |
| Okuno S 2019 | * | * | * | * | * | * | * | * | 8 |
| Ramanan B 2019 | * | * | * | * | * | * | - | * | 7 |
| Shannon AH 2019 | * | * | * | * | * | * | - | * | 7 |
| Simons JP 2019 | * | * | * | * | * | * | * | * | 8 |
| Vossen RJ 2019 | * | * | * | * | * | * | * | * | 8 |
| Bodewes TCF 2018 | * | * | * | * | * | * | - | * | 7 |
| Ito R 2018 | * | * | * | * | * | * | * | * | 8 |
| Meyer A 2018 | * | * | * | * | - | * | * | * | 7 |
| Mohapatra A 2018 | * | * | * | * | ** | * | * | * | 9 |
| Robinson WP 2018 | * | * | * | * | * | * | - | * | 7 |
| Stavroulakis K 2018 | * | * | * | * | * | - | * | * | 7 |
| Veraldi GF 2018 | * | * | * | * | * | * | * | * | 8 |
| Biasi L 2017 | * | * | * | * | * | * | * | * | 8 |
| Chen SL 2017 | * | * | * | * | * | * | - | * | 7 |
| Darling JD 2017 | * | * | * | * | * | * | * | * | 8 |
| Davies MG 2017 | * | * | * | * | * | * | * | * | 8 |
| Iida O 2017 | * | * | * | * | * | * | * | * | 8 |
| Mehaffey JH 2017 | * | * | * | * | * | * | - | * | 7 |
| Morisaki K 2017 | * | * | * | * | * | * | * | * | 8 |
| Shiraki T 2017 | * | * | * | * | * | * | * | * | 8 |
| Bisdas T 2016 | * | * | * | * | * | * | * | * | 8 |
| Gentile F 2016 | * | * | * | * | * | * | * | * | 8 |
| Hicks CW 2016 | * | * | * | * | * | - | * | * | 7 |
| Inoue K 2016 | * | * | * | * | - | * | * | * | 7 |
| Lejay A 2016 | * | * | * | * | * | * | * | - | 7 |
| Meltzer AJ 2016 | * | * | * | * | * | * | - | * | 7 |
| Meyer A 2016 | * | * | * | * | * | * | * | - | 7 |
| Patel SD 2016 | * | * | * | * | * | * | * | * | 8 |
| Sigterman TA 2016 | * | * | * | * | * | * | - | * | 7 |
| Siracuse JJ 2016 | * | * | * | * | * | - | * | * | 7 |
| Bisdas T 2015 | * | * | * | - | * | * | * | * | 7 |
| Fallon JM 2015 | * | * | * | * | * | * | * | * | 8 |
| Jens S 2015 | * | * | * | * | - | - | * | * | 6 |
| Katib N 2015 | * | * | * | * | * | * | * | * | 8 |
| Ohmine T 2015 | * | * | * | * | * | * | * | * | 8 |
| Tsai TT 2015 | * | * | * | * | * | * | * | * | 8 |
| Islam J 2015 | * | * | * | * | * | * | * | * | 8 |
| Shiraki T 2014 | * | * | * | * | * | * | * | * | 8 |
| Garg K 2014 | * | * | * | * | * | * | * | - | 7 |
| Masaki H 2014 | * | * | * | * | * | * | * | * | 8 |
| Soga Y 2014 | * | * | * | * | * | * | * | * | 8 |
| Aihara H 2014 | * | - | * | * | * | * | * | * | 7 |
| Linnakoski H 2013 | * | - | * | * | * | * | * | - | 6 |
| Dosluoglu HH 2012 | * | * | * | * | * | * | * | - | 7 |
| Siracuse JJ 2012 | * | * | * | * | * | - | * | * | 7 |
| Faglia E 2012 | * | * | * | * | * | - | * | * | 7 |
| Korhonen M 2011 | * | * | * | * | ** | * | * | * | 9 |
| Arvela E 2011 | * | * | * | * | ** | * | * | * | 9 |
| Scali ST 2011 | * | - | * | * | * | * | * | * | 7 |
| Varela C 2011 | * | * | * | * | ** | * | * | * | 9 |
| Casella IB 2010 | * | - | * | * | ** | * | * | * | 8 |
| Soderstrom MI 2010 | * | * | * | * | ** | * | * | * | 9 |
| Ah Chong AK 2009 | * | * | * | * | * | * | * | - | 7 |
| Sultan S 2009 | * | * | * | * | * | * | * | * | 8 |
| Dick F 2007 | * | * | * | * | * | * | * | - | 7 |
| Kudo T 2006 | * | * | * | * | * | * | * | * | 8 |
| Faglia E 2006 | * | * | * | * | * | * | * | * | 8 |
| Taylor SM 2005 | * | * | * | * | - | * | * | - | 6 |
| Hynes N 2004 | * | * | * | * | - | * | * | * | 7 |

**Supplemental Table S4. The results of meta- regression**

| Outcomes | Estimate | | SE | *Z* | *P* |
| --- | --- | --- | --- | --- | --- |
|  | Coefficient | 95%CI |  |  |  |
| 30-day mortality |  |  |  |  |  |
| Study design | -0.17 | (-0.87, 0.52) | 0.35 | -0.49 | 0.622 |
| Multicenter | -0.44 | (-1.00, 0.12) | 0.29 | -1.54 | 0.124 |
| Regional characteristics | **-0.45** | **(-0.78, -0.13)** | **0.16** | **-2.78** | **0.006** |
| Sample size | 0.47 | (-0.05, 1.00) | 0.26 | 1.78 | 0.075 |
| Disease stage | -0.11 | (-0.46, 0.24) | 0.18 | -0.63 | 0.526 |
| Overall survival |  |  |  |  |  |
| Study design | 0.01 | (-0.27, 0.27) | 0.14 | 0.01 | 0.998 |
| Multicenter | -0.07 | (-0.20, 0.06) | 0.07 | -1.12 | 0.261 |
| Regional characteristics | **0.12** | **(0.19, 0.22)** | **0.05** | **2.35** | **0.018** |
| Sample size | -0.19 | (-0.31, -0.07) | 0.06 | -3.06 | 0.002 |
| Disease stage | -0.17 | (-0.32, -0.03) | 0.73 | -2.34 | 0.019 |

CI, confidence interval; SE, standard error.

**Supplemental Figure S1. The distribution of included studies in published year, geographical location.**

**Supplemental Figure S2. Risk of bias assessment of the included RCTs.**


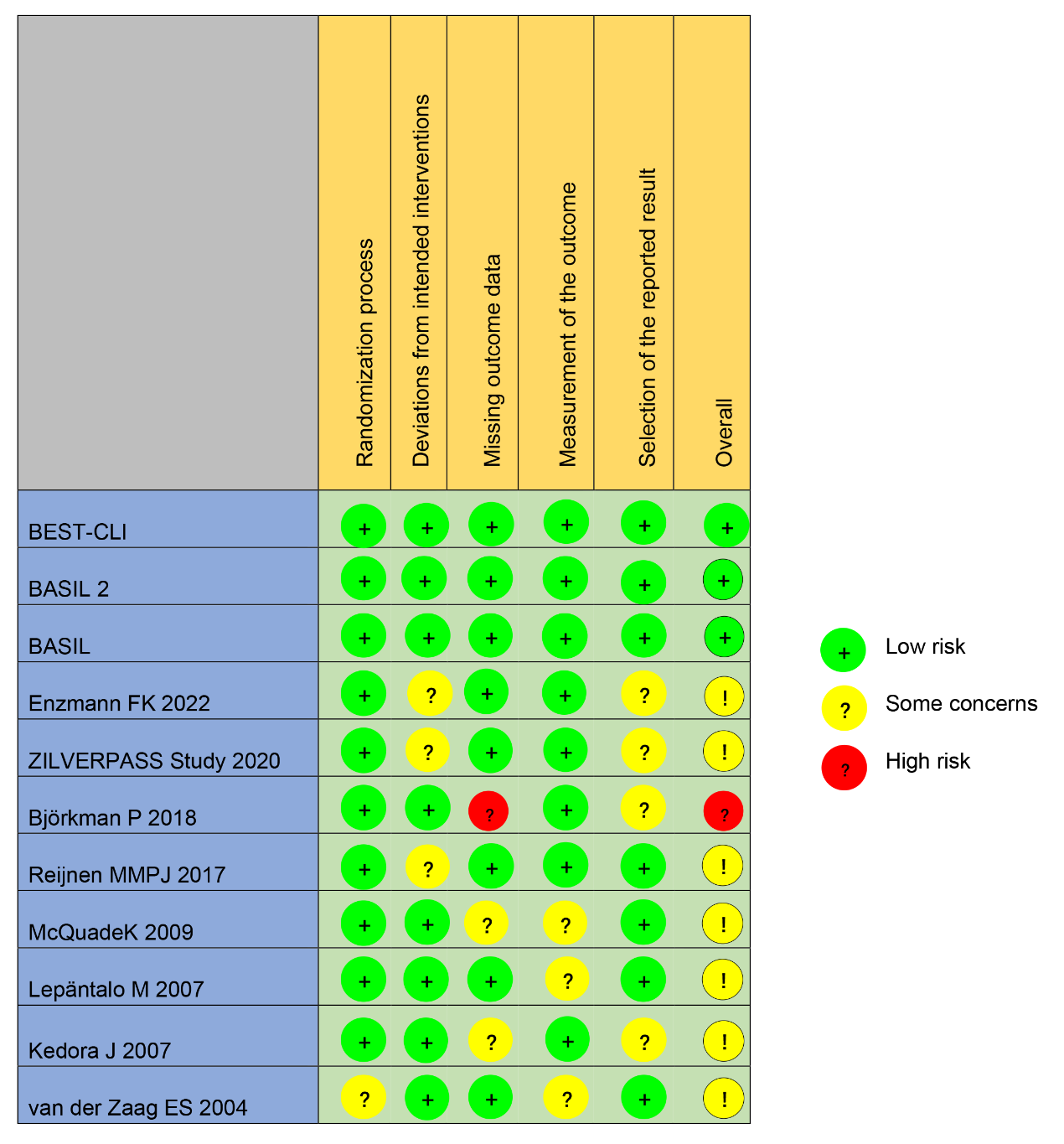


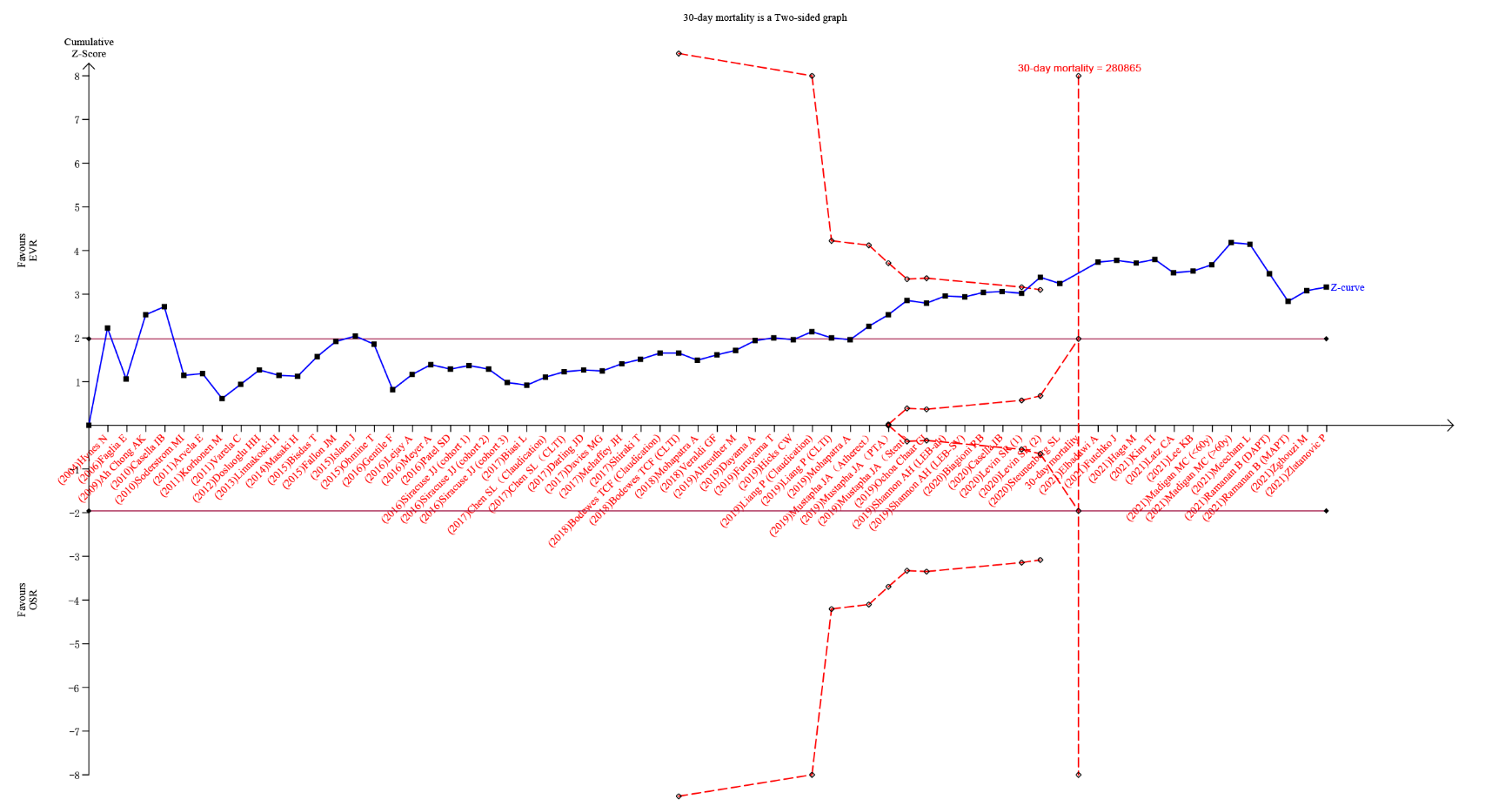
**Supplemental Figure S3. The trial sequential analysis results of 30-day mortality.**

The required information size was calculated based on a two-side α = 5%, power 80%, and a relative risk reduction of 20%.


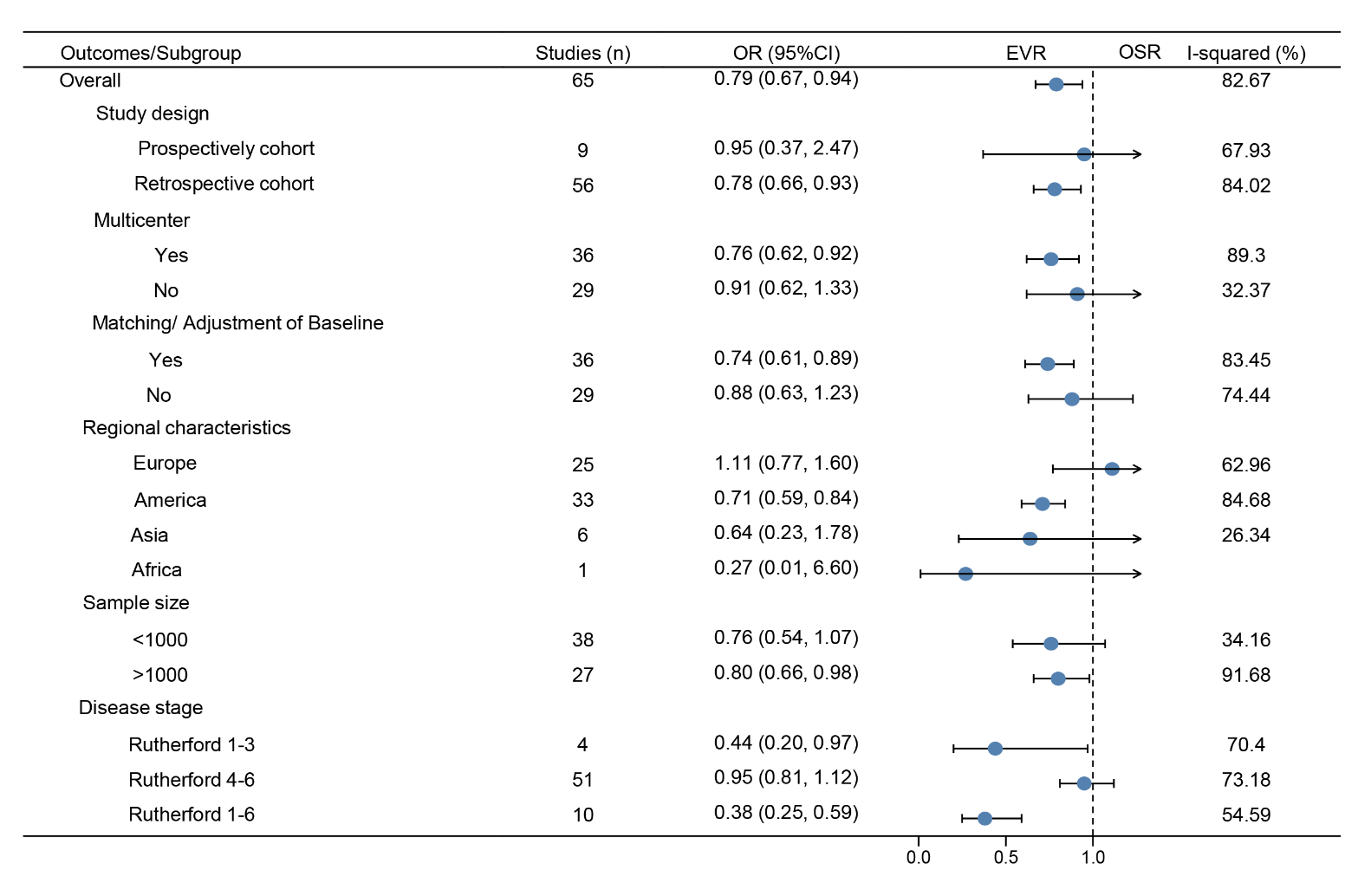
**Supplemental Figure S4. Subgroup analysis of 30-day mortality**

OR, odds ratio; EVR, endovascular revascularization; OSR, open surgical revascularization; CI, confidence interval.


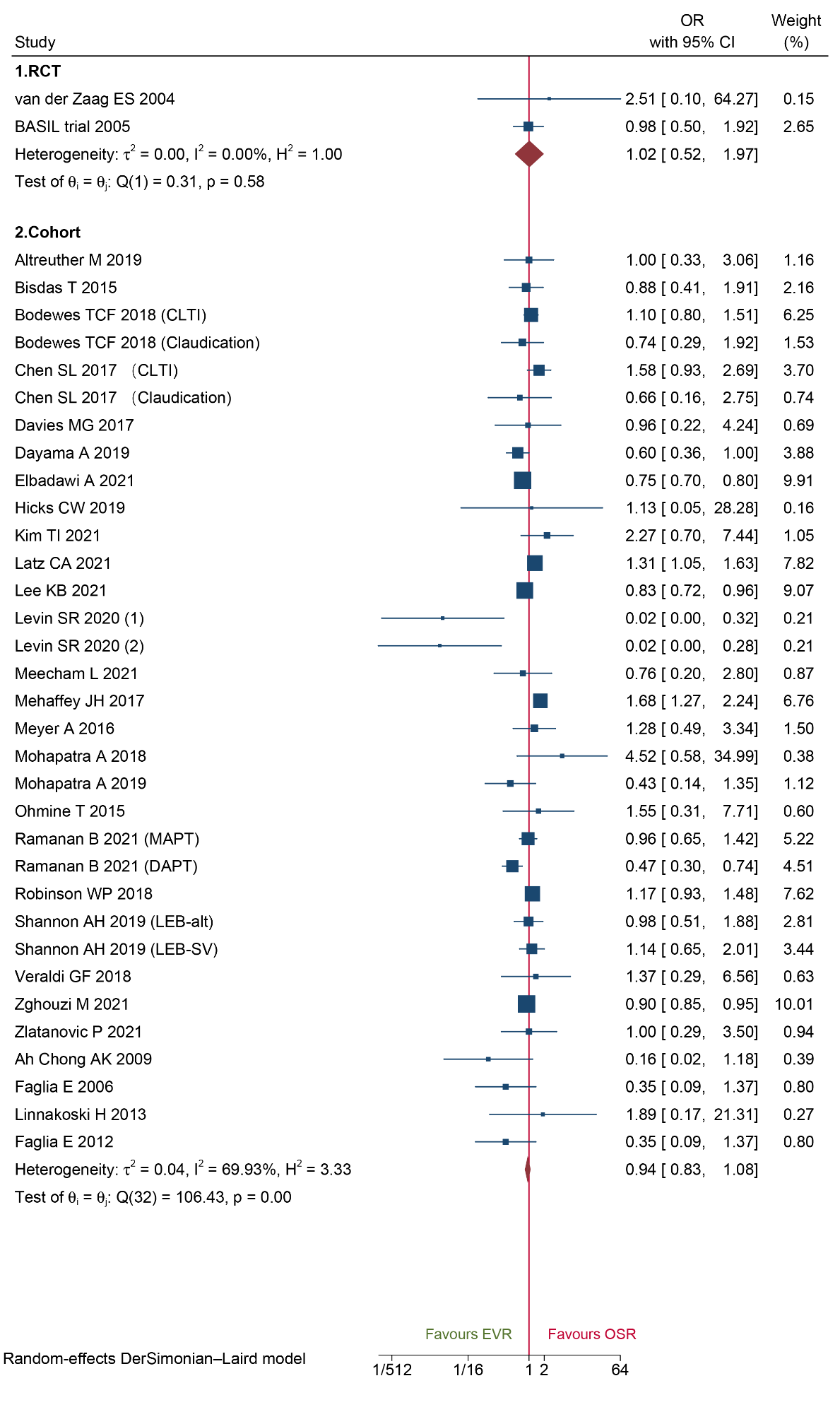
**Supplemental Figure S5. Pooled results of 30-day major amputation**

OR, odds ratio; EVR, endovascular revascularization; OSR, open surgical revascularization; CI, confidence interval; RCT, randomized controlled trial; CLTI, chronic limb-threatening ischemia; MAPT, mono antiplatelet agent; DAPT, dual antiplatelet agent. Squares indicate the odds ratio, and horizontal lines represent 95% confidence intervals.

**Supplemental Figure S6. Pooled results of wound complication**


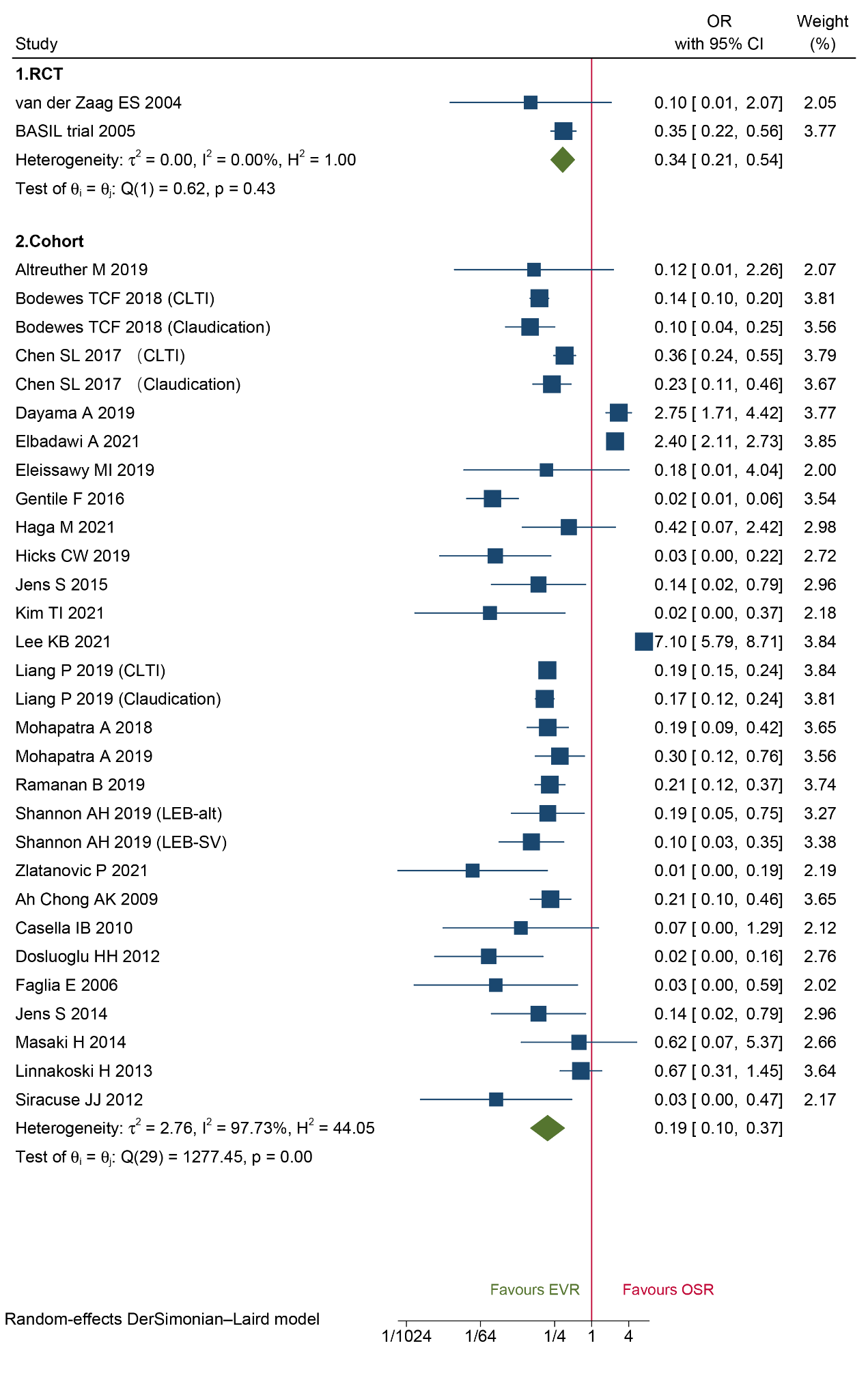


OR, odds ratio; EVR, endovascular revascularization; OSR, open surgical revascularization; CI, confidence interval; RCT, randomized controlled trial; CLTI, chronic limb-threatening ischemia; MAPT, mono antiplatelet agent; DAPT, dual antiplatelet agent. Squares indicate the odds ratio, and horizontal lines represent 95% confidence intervals.


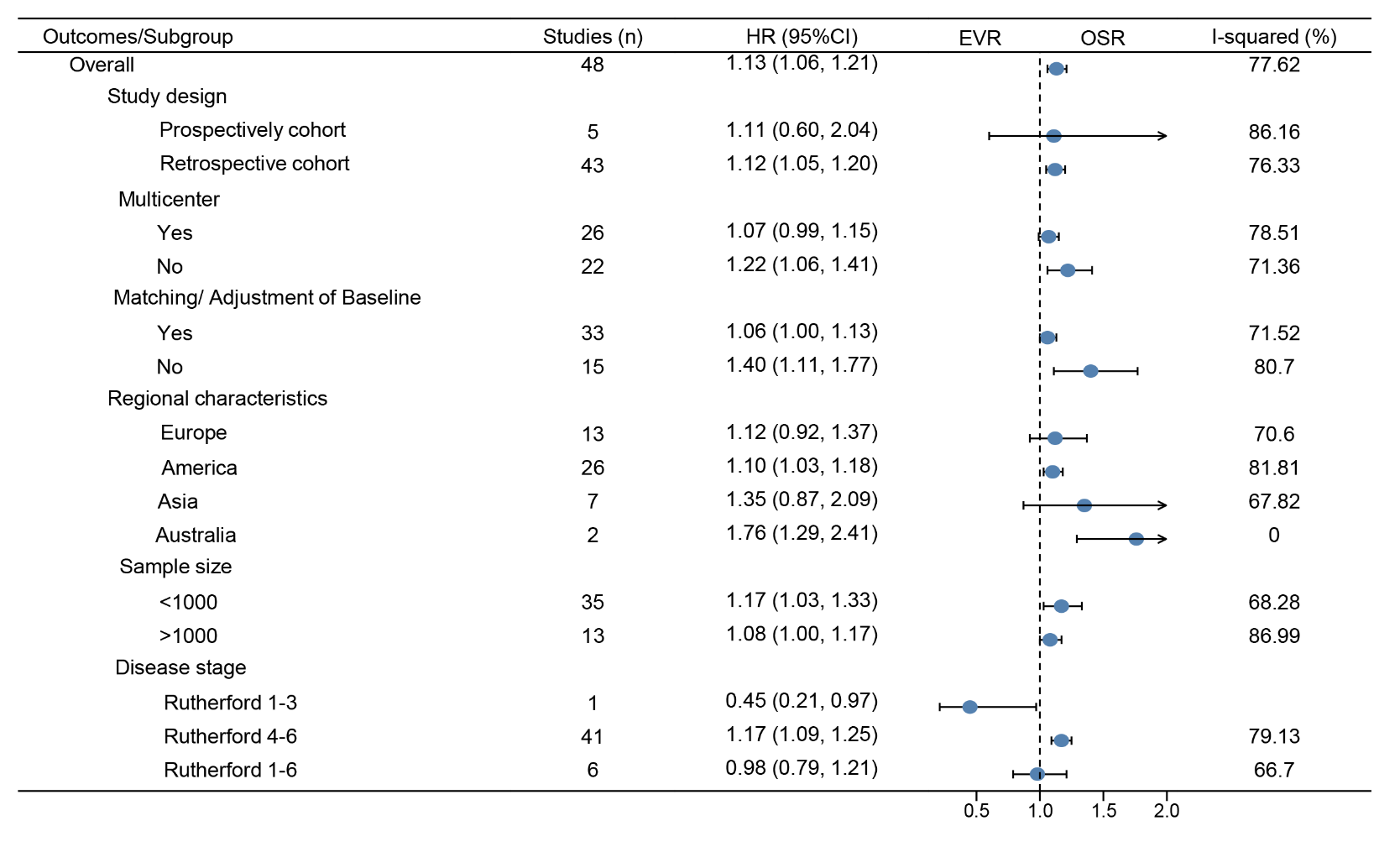
**Supplemental Figure S7. Subgroup analysis of overall survival**

OR, odds ratio; EVR, endovascular revascularization; OSR, open surgical revascularization; CI, confidence interval.

Supplemental References:

1. Bradbury AW, Moakes CA, Popplewell M, Meecham L, Bate GR, Kelly L, et al. A vein bypass first versus a best endovascular treatment first revascularisation strategy for patients with chronic limb threatening ischaemia who required an infra-popliteal, with or without an additional more proximal infra-inguinal revascularisation procedure to restore limb perfusion (BASIL-2): an open-label, randomised, multicentre, phase 3 trial. Lancet. 2023;401(10390):1798-809.

2. Farber A, Menard MT, Conte MS, Kaufman JA, Powell RJ, Choudhry NK, et al. Surgery or Endovascular Therapy for Chronic Limb-Threatening Ischemia. N Engl J Med. 2022;387(25):2305-16.

3. Enzmann FK, Nierlich P, Hölzenbein T, Aspalter M, Kluckner M, Hitzl W, et al. Vein Bypass versus Nitinol Stent in Long Femoropopliteal Lesions: 4-Year Results of a Randomized Controlled Trial. Ann Surg. 2022.

4. Bosiers M, Setacci C, De Donato G, Torsello G, Silveira PG, Deloose K, et al. ZILVERPASS Study: ZILVER PTX Stent vs Bypass Surgery in Femoropopliteal Lesions. J Endovasc Ther. 2020;27(2):287-95.

5. Björkman P, Auvinen T, Hakovirta H, Romsi P, Turtiainen J, Manninen H, et al. Drug-Eluting Stent Shows Similar Patency Results as Prosthetic Bypass in Patients with Femoropopliteal Occlusion in a Randomized Trial. Ann Vasc Surg. 2018;53:165-70.

6. Reijnen M, van Walraven LA, Fritschy WM, Lensvelt MMA, Zeebregts CJ, Lemson MS, et al. 1-Year Results of a Multicenter Randomized Controlled Trial Comparing Heparin-Bonded Endoluminal to Femoropopliteal Bypass. JACC Cardiovasc Interv. 2017;10(22):2320-31.

7. McQuade K, Gable D, Hohman S, Pearl G, Theune B. Randomized comparison of ePTFE/nitinol self-expanding stent graft vs prosthetic femoral-popliteal bypass in the treatment of superficial femoral artery occlusive disease. J Vasc Surg. 2009;49(1):109-15, 16.e1-9; discussion 16.

8. Bradbury AW, Adam DJ, Bell J, Forbes JF, Fowkes FG, Gillespie I, et al. Bypass versus Angioplasty in Severe Ischaemia of the Leg (BASIL) trial: An intention-to-treat analysis of amputation-free and overall survival in patients randomized to a bypass surgery-first or a balloon angioplasty-first revascularization strategy. J Vasc Surg. 2010;51(5 Suppl):5s-17s.

9. Adam DJ, Beard JD, Cleveland T, Bell J, Bradbury AW, Forbes JF, et al. Bypass versus angioplasty in severe ischaemia of the leg (BASIL): multicentre, randomised controlled trial. Lancet. 2005;366(9501):1925-34.

10. Kedora J, Hohmann S, Garrett W, Munschaur C, Theune B, Gable D. Randomized comparison of percutaneous Viabahn stent grafts vs prosthetic femoral-popliteal bypass in the treatment of superficial femoral arterial occlusive disease. J Vasc Surg. 2007;45(1):10-6; discussion 6.

11. Lepäntalo M, Laurila K, Roth WD, Rossi P, Lavonen J, Mäkinen K, et al. PTFE bypass or thrupass for superficial femoral artery occlusion? A randomised controlled trial. Eur J Vasc Endovasc Surg. 2009;37(5):578-84.

12. van der Zaag ES, Legemate DA, Prins MH, Reekers JA, Jacobs MJ. Angioplasty or bypass for superficial femoral artery disease? A randomised controlled trial. Eur J Vasc Endovasc Surg. 2004;28(2):132-7.

13. Mathlouthi A, Elsayed N, Al-Nouri O, Farber A, Malas MB. Outcomes of Endovascular-First Versus Bypass-First Approach for Patients With Chronic Limb-Threatening Ischemia Using a Medicare-Linked Database. Ann Vasc Surg. 2022;85:119-24.

14. Lee CY. Clinical Effect of Revascularization Strategies and Pharmacologic Treatment on Long-Term Results in Patients with Advanced Peripheral Artery Disease with TASC C and D Femoropopliteal Lesions. J Interv Cardiol. 2022;2022:3741967.

15. Elbadawi A, Elgendy IY, Saad M, Elzeneini M, Megaly M, Omer M, et al. Contemporary Revascularization Strategies and Outcomes Among Patients With Diabetes With Critical Limb Ischemia: Insights From the National Inpatient Sample. JACC Cardiovasc Interv. 2021;14(6):664-74.

16. Futchko J, Friedmann P, Phair J, Trestman EB, Denesopolis J, Shariff S, et al. A Propensity-Matched Analysis of Endovascular Intervention versus Open Nonautologous Bypass as Initial Therapy in Patients with Chronic Limb-Threatening Ischemia. Ann Vasc Surg. 2021;75:194-204.

17. Haga M, Shindo S, Motohashi S, Nishiyama A, Kimura M, Inoue H, et al. Early evaluation of the infrainguinal revascularization strategy selection tool of the Global Vascular Guidelines for chronic limb-threatening ischemia patients. J Vasc Surg. 2021;74(4):1253-60.e2.

18. Kim TI, Zhang Y, Cardella JA, Guzman RJ, Ochoa Chaar CI. Outcomes of bypass and endovascular interventions for advanced femoropopliteal disease in patients with premature peripheral artery disease. J Vasc Surg. 2021;74(6):1968-77.e3.

19. Latz CA, Boitano L, Wang LJ, Pendleton AA, DeCarlo C, Sumpio B, et al. Contemporary Endovascular 30-Day Outcomes for Critical Limb Threatening Ischemia Relative to Surgical Bypass Grafting. Vasc Endovascular Surg. 2021;55(5):441-7.

20. Lee KB, Macsata RA, Lala S, Sparks AD, Amdur RL, Ricotta JJ, et al. Outcomes of open and endovascular interventions in patients with chronic limb threatening ischemia. Vascular. 2021;29(5):693-703.

21. Madigan MC, Farber A, Rybin DV, Doros G, Robinson WP, 3rd, Siracuse JJ, et al. Younger patients have worse outcomes after peripheral endovascular interventions for suprainguinal arterial occlusive disease. J Vasc Surg. 2021;73(5):1715-22.

22. Meecham L, Popplewell MA, Bate GR, Patel S, Bradbury AW. A Comparison of Contemporary Clinical Outcomes Following Femoro-Popliteal Plain Balloon Angioplasty and Bypass Surgery for Chronic Limb Threatening Ischemia. Vasc Endovascular Surg. 2021;55(6):544-50.

23. Ramanan B, Jeon-Slaughter H, Chen X, Kashyap VS, Kirkwood ML, Timaran CH, et al. Impact of dual antiplatelet therapy after lower extremity revascularization for chronic limb-threatening ischemia. J Vasc Surg. 2021;74(4):1327-34.

24. Zghouzi M, Moussa Pacha H, Ullah W, Sattar Y, Ahmad B, Osman H, et al. In-hospital outcomes of endovascular versus surgical revascularization for chronic total occlusion in peripheral artery disease. Catheter Cardiovasc Interv. 2021;98(4):E586-e93.

25. Zlatanovic P, Mahmoud AA, Cinara I, Cvetic V, Lukic B, Davidovic L. Comparison of Long Term Outcomes After Endovascular Treatment Versus Bypass Surgery in Chronic Limb Threatening Ischaemia Patients with Long Femoropopliteal Lesions. Eur J Vasc Endovasc Surg. 2021;61(2):258-69.

26. Biagioni RB, Nasser F, Matielo MF, Burihan MC, Brochado Neto FC, Ingrund JC, et al. Comparison of Bypass and Endovascular Intervention for Popliteal Occlusion with the Involvement of Trifurcation for Critical Limb Ischemia. Ann Vasc Surg. 2020;63:218-26.

27. Casella IB, Sartori CH, Faustino CB, Vieira Mariz MP, Presti C, Puech-Leão P, et al. Endovascular Therapy Provides Similar Results of Bypass Graft Surgery in the Treatment of Infrainguinal Multilevel Arterial Disease in Patients with Chronic Limb-Threatening Ischemia in All GLASS Stages. Ann Vasc Surg. 2020;68:400-8.

28. Lawaetz M, Fisker L, Lönn L, Sillesen H, Eiberg J. In Situ Vein Bypass Is Superior to Endovascular Treatment of Femoropopliteal Lesions in Chronic Limb-Threatening Ischemia. Ann Vasc Surg. 2020;67:437-47.

29. Levin SR, Farber A, Cheng TW, Arinze N, Jones DW, Rybin D, et al. Patients undergoing interventions for claudication experience low perioperative morbidity but are at risk for worsening functional status and limb loss. J Vasc Surg. 2020;72(1):241-9.

30. Perlander A, Jivegård L, Nordanstig J, Svensson M, Österberg K. Amputation-free survival, limb symptom alleviation, and reintervention rates after open and endovascular revascularization of femoropopliteal lesions in patients with chronic limb-threatening ischemia. J Vasc Surg. 2020;72(6):1987-95.

31. Steunenberg SL, de Vries J, Raats JW, Verbogt N, Lodder P, van Eijck GJ, et al. Quality of Life and Traditional Outcome Results at 1 Year in Elderly Patients Having Critical Limb Ischemia and the Role of Conservative Treatment. Vasc Endovascular Surg. 2020;54(2):126-34.

32. Altreuther M, Mattsson E. Long-Term Limb Salvage and Amputation-Free Survival After Femoropopliteal Bypass and Femoropopliteal PTA for Critical Ischemia in a Clinical Cohort. Vasc Endovascular Surg. 2019;53(2):112-7.

33. Dayama A, Tsilimparis N, Kolakowski S, Matolo NM, Humphries MD. Clinical outcomes of bypass-first versus endovascular-first strategy in patients with chronic limb-threatening ischemia due to infrageniculate arterial disease. J Vasc Surg. 2019;69(1):156-63.e1.

34. Eleissawy MI, Elbarbary AH, Elwagih MM, Elheniedy MA, Santoso C, Fourneau I. Ipsilateral Antegrade Angioplasty for Flush Superficial Femoral Artery Occlusion versus Open Bypass Surgery. Ann Vasc Surg. 2019;61:55-64.

35. Furuyama T, Onohara T, Yoshiga R, Yoshiya K, Matsubara Y, Inoue K, et al. Functional prognosis of critical limb ischemia and efficacy of restoration of direct flow below the ankle. Vascular. 2019;27(1):38-45.

36. Hicks CW, Canner JK, Lum YW, Black JH, 3rd, Abularrage CJ. Long-term Outcomes of an Endovascular-First Approach for Diabetic Patients With Predominantly Tibial Disease Treated in a Multidisciplinary Setting. Ann Vasc Surg. 2019;60:315-26.e2.

37. Liang P, Li C, O'Donnell TFX, Lo RC, Soden PA, Swerdlow NJ, et al. In-hospital versus postdischarge major adverse events within 30 days following lower extremity revascularization. J Vasc Surg. 2019;69(2):482-9.

38. Lin JH, Brunson A, Romano PS, Mell MW, Humphries MD. Endovascular-First Treatment Is Associated With Improved Amputation-Free Survival in Patients With Critical Limb Ischemia. Circ Cardiovasc Qual Outcomes. 2019;12(8):e005273.

39. Mohapatra A, Boitet A, Malak O, Henry JC, Avgerinos ED, Makaroun MS, et al. Peroneal bypass versus endovascular peroneal intervention for critical limb ischemia. J Vasc Surg. 2019;69(1):148-55.

40. Mustapha JA, Katzen BT, Neville RF, Lookstein RA, Zeller T, Miller LE, et al. Propensity Score-Adjusted Comparison of Long-Term Outcomes Among Revascularization Strategies for Critical Limb Ischemia. Circ Cardiovasc Interv. 2019;12(9):e008097.

41. Ochoa Chaar CI, Gholitabar N, Goodney P, Dardik A, Abougergi MS. One-Year Readmission after Open and Endovascular Revascularization for Critical Limb Ischemia. Ann Vasc Surg. 2019;61:25-32.e2.

42. Okuno S, Iida O, Iida T, Takahara M, Yamaoka T, Kitano I, et al. Comparison of Clinical Outcomes between Endovascular Therapy with Self-Expandable Nitinol Stent and Femoral-Popliteal Bypass for Trans-Atlantic Inter-Society Consensus II C and D Femoropopliteal Lesions. Ann Vasc Surg. 2019;57:137-43.

43. Ramanan B, Jeon-Slaughter H, Chen X, Modrall JG, Tsai S. Comparison of open and endovascular procedures in patients with critical limb ischemia on dialysis. J Vasc Surg. 2019;70(4):1217-24.

44. Shannon AH, Mehaffey JH, Cullen JM, Upchurch GR, Jr., Robinson WP, 3rd. A Comparison of Outcomes After Lower Extremity Bypass and Repeat Endovascular Intervention Following Failed Previous Endovascular Intervention for Critical Limb Ischemia. Angiology. 2019;70(6):501-5.

45. Simons JP, Schanzer A, Flahive JM, Osborne NH, Mills JL, Sr., Bradbury AW, et al. Survival prediction in patients with chronic limb-threatening ischemia who undergo infrainguinal revascularization. J Vasc Surg. 2019;69(6s):137S-51S.e3.

46. Vossen RJ, Vahl AC, Fokkema TM, Leijdekkers VJ, van Swijndregt AM, Balm R. Endovascular therapy versus femoropopliteal bypass surgery for medium-length TASC II B and C lesions of the superficial femoral artery: An observational propensity-matched analysis. Vascular. 2019;27(5):542-52.

47. Bodewes TCF, Darling JD, Deery SE, O'Donnell TFX, Pothof AB, Shean KE, et al. Patient selection and perioperative outcomes of bypass and endovascular intervention as first revascularization strategy for infrainguinal arterial disease. J Vasc Surg. 2018;67(1):206-16.e2.

48. Ito R, Kumada Y, Ishii H, Kamoi D, Sakakibara T, Umemoto N, et al. Clinical Outcomes after Isolated Infrapopliteal Revascularization in Hemodialysis Patients with Critical Limb Ischemia: Endovascular Therapy versus Bypass Surgery. J Atheroscler Thromb. 2018;25(9):799-807.

49. Meyer A, Schilling A, Kott M, Rother U, Lang W, Regus S. Open Versus Endovascular Revascularization of Below-Knee Arteries in Patients With End-Stage Renal Disease and Critical Limb Ischemia. Vasc Endovascular Surg. 2018;52(8):613-20.

50. Mohapatra A, Henry JC, Avgerinos ED, Boitet A, Chaer RA, Makaroun MS, et al. Bypass versus endovascular intervention for healing ischemic foot wounds secondary to tibial arterial disease. J Vasc Surg. 2018;68(1):168-75.

51. Robinson WP, Mehaffey JH, Hawkins RB, Tracci MC, Cherry KJ, Eslami M, et al. Lower extremity bypass and endovascular intervention for critical limb ischemia fail to meet Society for Vascular Surgery's objective performance goals for limb-related outcomes in a contemporary national cohort. J Vasc Surg. 2018;68(5):1438-45.

52. Stavroulakis K, Borowski M, Torsello G, Bisdas T. One-Year Results of First-Line Treatment Strategies in Patients With Critical Limb Ischemia (CRITISCH Registry). J Endovasc Ther. 2018;25(3):320-9.

53. Veraldi GF, Mezzetto L, Macrì M, Criscenti P, Corvasce A, Poli R. Comparison of Endovascular Versus Bypass Surgery in Femoropopliteal TASC II D Lesions: A Single-Center Study. Ann Vasc Surg. 2018;47:179-87.

54. Biasi L, Patel SD, Lea T, Donati T, Katsanos K, Partridge JS, et al. Complex infrapopliteal revascularization in elderly patients with critical limb ischemia: impact of multidisciplinary integrated care on mid-term outcome. J Cardiovasc Surg (Torino). 2017;58(5):665-73.

55. Chen SL, Whealon MD, Kabutey NK, Kuo IJ, Sgroi MD, Fujitani RM. Outcomes of open and endovascular lower extremity revascularization in active smokers with advanced peripheral arterial disease. J Vasc Surg. 2017;65(6):1680-9.

56. Darling JD, McCallum JC, Soden PA, Korepta L, Guzman RJ, Wyers MC, et al. Results for primary bypass versus primary angioplasty/stent for lower extremity chronic limb-threatening ischemia. J Vasc Surg. 2017;66(2):466-75.

57. Davies MG, El-Sayed HF. Outcomes of native superficial femoral artery chronic total occlusion recanalization after failed femoropopliteal bypass. J Vasc Surg. 2017;65(3):726-33.

58. Iida O, Takahara M, Soga Y, Kodama A, Terashi H, Azuma N. Three-Year Outcomes of Surgical Versus Endovascular Revascularization for Critical Limb Ischemia: The SPINACH Study (Surgical Reconstruction Versus Peripheral Intervention in Patients With Critical Limb Ischemia). Circ Cardiovasc Interv. 2017;10(12):e005531.

59. Mehaffey JH, Hawkins RB, Fashandi A, Cherry KJ, Kern JA, Kron IL, et al. Lower extremity bypass for critical limb ischemia decreases major adverse limb events with equivalent cardiac risk compared with endovascular intervention. J Vasc Surg. 2017;66(4):1109-16.e1.

60. Morisaki K, Matsumoto T, Matsubara Y, Inoue K, Aoyagi Y, Matsuda D, et al. Prognostic factor of the two-year mortality after revascularization in patients with critical limb ischemia. Vascular. 2017;25(2):123-9.

61. Shiraki T, Iida O, Takahara M, Soga Y, Mii S, Okazaki J, et al. Comparison of Clinical Outcomes after Surgical and Endovascular Revascularization in Hemodialysis Patients with Critical Limb Ischemia. J Atheroscler Thromb. 2017;24(6):621-9.

62. Bisdas T, Borowski M, Stavroulakis K, Torsello G. Endovascular Therapy Versus Bypass Surgery as First-Line Treatment Strategies for Critical Limb Ischemia: Results of the Interim Analysis of the CRITISCH Registry. JACC Cardiovasc Interv. 2016;9(24):2557-65.

63. Gentile F, Lundberg G, Hultgren R. Outcome for Endovascular and Open Procedures in Infrapopliteal Lesions for Critical Limb Ischemia: Registry Based Single Center Study. Eur J Vasc Endovasc Surg. 2016;52(5):643-9.

64. Hicks CW, Najafian A, Farber A, Menard MT, Malas MB, Black JH, 3rd, et al. Diabetes does not worsen outcomes following infrageniculate bypass or endovascular intervention for patients with critical limb ischemia. J Vasc Surg. 2016;64(6):1667-74.e1.

65. Inoue K, Onohara T, Mikasa K, Furuyama T. Early-phase wound healing and long-term outcomes of a selective endovascular-first approach for treating Rutherford 5 critical limb ischemia with infrainguinal lesions. Surg Today. 2016;46(11):1301-9.

66. Lejay A, Delay C, Georg Y, Schwein A, Gaertner S, Thaveau F, et al. Endovascular Surgery, Open Surgery, and Primary Amputation in Nonagenarians Presenting with Critical Limb Ischemia. Ann Vasc Surg. 2016;32:25-33.

67. Meltzer AJ, Sedrakyan A, Isaacs A, Connolly PH, Schneider DB. Comparative effectiveness of peripheral vascular intervention versus surgical bypass for critical limb ischemia in the Vascular Study Group of Greater New York. J Vasc Surg. 2016;64(5):1320-6.e2.

68. Meyer A, Lang W, Borowski M, Torsello G, Bisdas T. In-hospital outcomes in patients with critical limb ischemia and end-stage renal disease after revascularization. J Vasc Surg. 2016;63(4):966-73.

69. Patel SD, Biasi L, Paraskevopoulos I, Silickas J, Lea T, Diamantopoulos A, et al. Comparison of angioplasty and bypass surgery for critical limb ischaemia in patients with infrapopliteal peripheral artery disease. Br J Surg. 2016;103(13):1815-22.

70. Sigterman TA, Bolt LJ, Krasznai AG, Snoeijs MG, Heijboer R, Schurink GH, et al. Loss of kidney function in patients with critical limb ischemia treated endovascularly or surgically. J Vasc Surg. 2016;64(2):362-8.

71. Siracuse JJ, Menard MT, Eslami MH, Kalish JA, Robinson WP, Eberhardt RT, et al. Comparison of open and endovascular treatment of patients with critical limb ischemia in the Vascular Quality Initiative. J Vasc Surg. 2016;63(4):958-65.e1.

72. Bisdas T, Borowski M, Torsello G. Current practice of first-line treatment strategies in patients with critical limb ischemia. J Vasc Surg. 2015;62(4):965-73.e3.

73. Fallon JM, Goodney PP, Stone DH, Patel VI, Nolan BW, Kalish JA, et al. Outcomes of lower extremity revascularization among the hemodialysis-dependent. J Vasc Surg. 2015;62(5):1183-91.e1.

74. Jens S, Conijn AP, Frans FA, Nieuwenhuis MB, Met R, Koelemay MJ, et al. Outcomes of infrainguinal revascularizations with endovascular first strategy in critical limb ischemia. Cardiovasc Intervent Radiol. 2015;38(3):552-9.

75. Katib N, Thomas SD, Lennox AF, Yang JL, Varcoe RL. An Endovascular-First Approach to the Treatment of Critical Limb Ischemia Results in Superior Limb Salvage Rates. J Endovasc Ther. 2015;22(4):473-81.

76. Ohmine T, Iwasa K, Yamaoka T. Strategy of Revascularization for Critical Limb Ischemia Due to Infragenicular Lesions-Which Should Be Selected Firstly, Bypass Surgery or Endovascular Therapy? Ann Vasc Dis. 2015;8(4):275-81.

77. Tsai TT, Rehring TF, Rogers RK, Shetterly SM, Wagner NM, Gupta R, et al. The Contemporary Safety and Effectiveness of Lower Extremity Bypass Surgery and Peripheral Endovascular Interventions in the Treatment of Symptomatic Peripheral Arterial Disease. Circulation. 2015;132(21):1999-2011.

78. Islam J, Robbs JV. Comparison between superficial femoral artery stenting and bypass surgery in severe lower-limb ischaemia: a retrospective study. Cardiovasc J Afr. 2015;26(1):34-7.

79. Shiraki T, Iida O, Takahara M, Okamoto S, Kitano I, Tsuji Y, et al. Predictive scoring model of mortality after surgical or endovascular revascularization in patients with critical limb ischemia. J Vasc Surg. 2014;60(2):383-9.

80. Garg K, Kaszubski PA, Moridzadeh R, Rockman CB, Adelman MA, Maldonado TS, et al. Endovascular-first approach is not associated with worse amputation-free survival in appropriately selected patients with critical limb ischemia. J Vasc Surg. 2014;59(2):392-9.

81. Masaki H, Tabuchi A, Yunoki Y, Watanabe Y, Mimura D, Furukawa H, et al. Bypass vs. Endovascular Therapy of Infrapopliteal Lesions for Critical Limb Ischemia. Ann Vasc Dis. 2014;7(3):227-31.

82. Soga Y, Mii S, Iida O, Okazaki J, Kuma S, Hirano K, et al. Propensity score analysis of clinical outcome after bypass surgery vs. endovascular therapy for infrainguinal artery disease in patients with critical limb ischemia. J Endovasc Ther. 2014;21(2):243-53.

83. Aihara H, Soga Y, Mii S, Okazaki J, Yamaoka T, Kamoi D, et al. Comparison of long-term outcome after endovascular therapy versus bypass surgery in claudication patients with Trans-Atlantic Inter-Society Consensus-II C and D femoropopliteal disease. Circ J. 2014;78(2):457-64.

84. Linnakoski H, Uurto I, Suominen V, Vakhitov D, Salenius J. Comparison of above-the-knee prosthetic femoro-popliteal bypass versus percutaneous transluminal angioplasty and stenting for treatment of occlusive superficial femoral artery disease. Scand J Surg. 2013;102(4):227-33.

85. Dosluoglu HH, Lall P, Harris LM, Dryjski ML. Long-term limb salvage and survival after endovascular and open revascularization for critical limb ischemia after adoption of endovascular-first approach by vascular surgeons. J Vasc Surg. 2012;56(2):361-71.

86. Siracuse JJ, Giles KA, Pomposelli FB, Hamdan AD, Wyers MC, Chaikof EL, et al. Results for primary bypass versus primary angioplasty/stent for intermittent claudication due to superficial femoral artery occlusive disease. J Vasc Surg. 2012;55(4):1001-7.

87. Faglia E, Clerici G, Losa S, Tavano D, Caminiti M, Miramonti M, et al. Limb revascularization feasibility in diabetic patients with critical limb ischemia: results from a cohort of 344 consecutive unselected diabetic patients evaluated in 2009. Diabetes Res Clin Pract. 2012;95(3):364-71.

88. Korhonen M, Biancari F, Söderström M, Arvela E, Halmesmäki K, Albäck A, et al. Femoropopliteal balloon angioplasty vs. bypass surgery for CLI: a propensity score analysis. Eur J Vasc Endovasc Surg. 2011;41(3):378-84.

89. Arvela E, Venermo M, Söderström M, Korhonen M, Halmesmäki K, Albäck A, et al. Infrainguinal percutaneous transluminal angioplasty or bypass surgery in patients aged 80 years and older with critical leg ischaemia. Br J Surg. 2011;98(4):518-26.

90. Scali ST, Rzucidlo EM, Bjerke AA, Stone DH, Walsh DB, Goodney PP, et al. Long-term results of open and endovascular revascularization of superficial femoral artery occlusive disease. J Vasc Surg. 2011;54(3):714-21.

91. Varela C, Acin F, De Haro J, March J, Florez A, Lopez-Quintana A. Influence of surgical or endovascular distal revascularization of the lower limbs on ischemic ulcer healing. J Cardiovasc Surg (Torino). 2011;52(3):381-9.

92. Casella IB, Brochado-Neto FC, Sandri Gde A, Kalaf MJ, Godoy MR, Costa VS, et al. Outcome analysis of infrapopliteal percutaneous transluminal angioplasty and bypass graft surgery with nonreversed saphenous vein for individuals with critical limb ischemia. Vasc Endovascular Surg. 2010;44(8):625-32.

93. Söderström MI, Arvela EM, Korhonen M, Halmesmäki KH, Albäck AN, Biancari F, et al. Infrapopliteal percutaneous transluminal angioplasty versus bypass surgery as first-line strategies in critical leg ischemia: a propensity score analysis. Ann Surg. 2010;252(5):765-73.

94. Ah Chong AK, Tan CB, Wong MW, Cheng FS. Bypass surgery or percutaneous transluminal angioplasty to treat critical lower limb ischaemia due to infrainguinal arterial occlusive disease? Hong Kong Med J. 2009;15(4):249-54.

95. Sultan S, Hynes N. Five-year Irish trial of CLI patients with TASC II type C/D lesions undergoing subintimal angioplasty or bypass surgery based on plaque echolucency. J Endovasc Ther. 2009;16(3):270-83.

96. Dick F, Diehm N, Galimanis A, Husmann M, Schmidli J, Baumgartner I. Surgical or endovascular revascularization in patients with critical limb ischemia: influence of diabetes mellitus on clinical outcome. J Vasc Surg. 2007;45(4):751-61.

97. Kudo T, Chandra FA, Kwun WH, Haas BT, Ahn SS. Changing pattern of surgical revascularization for critical limb ischemia over 12 years: endovascular vs. open bypass surgery. J Vasc Surg. 2006;44(2):304-13.

98. Faglia E, Clerici G, Clerissi J, Gabrielli L, Losa S, Mantero M, et al. Early and five-year amputation and survival rate of diabetic patients with critical limb ischemia: data of a cohort study of 564 patients. Eur J Vasc Endovasc Surg. 2006;32(5):484-90.

99. Taylor SM, Kalbaugh CA, Blackhurst DW, Langan EM, 3rd, Cull DL, Snyder BA, et al. Postoperative outcomes according to preoperative medical and functional status after infrainguinal revascularization for critical limb ischemia in patients 80 years and older. Am Surg. 2005;71(8):640-5; discussion 5-6.

100. Hynes N, Akhtar Y, Manning B, Aremu M, Oiakhinan K, Courtney D, et al. Subintimal angioplasty as a primary modality in the management of critical limb ischemia: comparison to bypass grafting for aortoiliac and femoropopliteal occlusive disease. J Endovasc Ther. 2004;11(4):460-71.
